# Structure–Activity Relationship of Phytotoxic Natural 10-Membered Lactones and Their Semisynthetic Derivatives

**DOI:** 10.3390/jof7100829

**Published:** 2021-10-03

**Authors:** Anna Dalinova, Anatoly Fedorov, Vsevolod Dubovik, Olga Voitsekhovskaja, Elena Tyutereva, Sergey Smirnov, Dmitry Kochura, Leonid Chisty, Igor Senderskiy, Alexander Berestetskiy

**Affiliations:** 1 All-Russian Institute of Plant Protection, Saint Petersburg 196608, Russia; afedorov@vizr.spb.ru (A.F.); vdubovik@vizr.spb.ru (V.D.); senderskiy@mail.ru (I.S.); aberestetskiy@vizr.spb.ru (A.B.); 2 Komarov Botanical Institute, Russian Academy of Sciences, Saint Petersburg 197376, Russia; ovoitse@binran.ru (O.V.); tuterlena@mail.ru (E.T.); 3 The Research Resources Center for Magnetic Resonance, St. Petersburg State University, Saint Petersburg 198504, Russia; sergey.smirnov@spbu.ru; 4 Research Institute of Hygiene, Occupational Pathology and Human Ecology, Federal Medical Biological Agency, Saint Petersburg 188663, Russia; 89117050635@yandex.ru (D.K.); mehrn.q2@gmail.com (L.C.)

**Keywords:** stagonolide, herbarumin, nonenolide, phytotoxicity bioassay, natural product-derived herbicide

## Abstract

Ten-membered lactones (nonenolides) demonstrate phytotoxic, antimicrobial, and fungicidal activity promising for the development of natural product-derived pesticides. The fungus *Stagonospora cirsii* is able to produce phytotoxic stagonolides A (**1**), J (**2**), K (**3**) and herbarumin I (**4**) with high yield. The aim of this study was to create a set of structurally related nonenolides and to reveal the structural features that affect their biological activity. Stagonolide A (**1**) and C-7 oxidized stagonolide K (**11**) showed the highest phytotoxicity in leaf puncture assay and agar seedlings assay. The oxidation of C-7 hydroxyl group (as in **1**, acetylstagonolide A (**10**) and (**11**) led to the manifestation of toxicity to microalgae, *Bacillus subtilis* and Sf9 cells regardless of the configuration of C-9 propyl chains (*R* in **1** and **10**, *S* in **11**). C-7 non-oxidized nonenolides displayed none or little non-target activity. Notably, 7*S* compounds were more phytotoxic than their 7*R* analogues. Due to the high inhibitory activity against seedling growth and the lack of side toxicity, mono- and bis(acetyl)- derivatives of herbarumin I were shown to be potent for the development of pre-emergent herbicides. The identified structural features can be used for the rational design of new herbicides.

## 1. Introduction

Secondary metabolites of microorganisms are structurally “optimized” by evolution to serve particular biological functions, including competition with other organisms. Therefore, characterization of these natural products can provide new scaffolds for the development of pesticides. Many active ingredients of conventional pesticides (i.e., kresoxim-methyl, azoxystrobin, kasugamycin and others) are the examples of such natural product-derived molecules [1]. However, the spread of pesticide-resistant populations of weeds and pests is far ahead of the pace of new pesticide development [2,3]. Thus, it is necessary to screen natural product libraries for the compounds that possess promising activity against weeds and pests. However, the lead molecules should combine high target activity with low side toxicity [2]. Therefore, detailed investigation of their biological properties and structure–activity relationships (SAR) is necessary for the screening of target-specific toxins for the development of natural product-derived pesticides.

**Figure 1:**
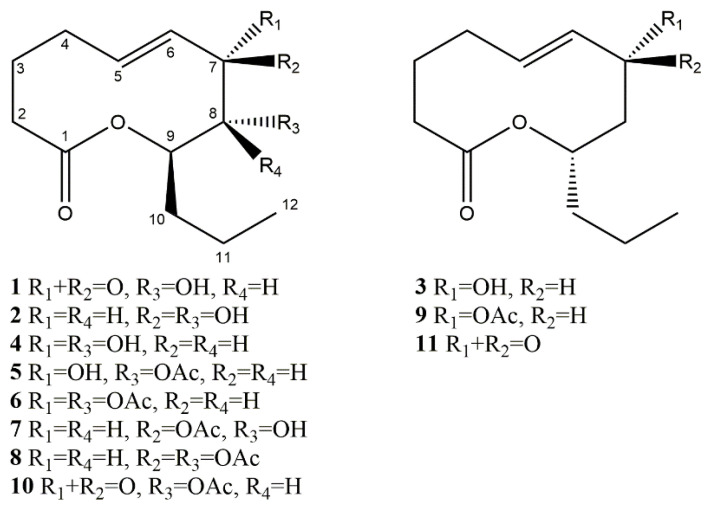
Natural nonenolides from *S. cirsii* (stagonolides A (**1**), J (**2**), K (**3**), herbarumin I (**4**)) and their semi-synthetic derivatives: 8-acetylherbarumin I (**5**), bis(acetyl)herbarumin I (**6**), 7-acetylstagonolide J (**7**), bis(acetyl)stagonolide J (**8**), acetylstagonolide K (**9**), 8-acetylstagonolide A (**10**), C-7 oxidized stagonolide K (**11**).

Ten-membered lactones (nonanolides, nonenolides, decanolides) are a common class of natural compounds that are produced by some fungi from *Pezizomycotina* such as *Phoma herbarum*, *Xylaria multiplex*, *Curvularia* sp., *Diplodia pinea*, *Stagonospora cirsii* and others [4,5,6,7,8,9,10]. Diverse members of this class of natural products have a common ten-membered lactone core and differ in the length of the alkyl chain at C9 and by the presence, position and configuration of substituents at C-2−C-8 (Figure 1) [11]. These molecules attract the attention of researchers, since they have a relatively simple structure and display a wide range of biological activities [11,12,13]. Some nonenolides (putaminoxins, herbarumins, stagonolides and others) are known as potent phytotoxins promising for the development of natural product-derived herbicides [10,14,15,16,17].

In our previous studies we have shown that the phytopathogenic pycnidial fungus *Stagonospora cirsii* Davis is a “biofactory” for nonenolides production with diverse structures and biological activities including stagonolides A−K, herbarumin I and modiolide A [10,15,16,17]. Strain G-51 *S. cirsii*, a specialized pathogen of perennial sowthistle (*Sonchus arvensis*), was patented as a producer of stagonolide A and herbarumin I at a preindustrial level (over 100 mg/L and 450 mg/kg, respectively) [18]. Among *S. cirsii* secondary metabolites, stagonolides A, J, K, and herbarumin I displayed promising phytotoxic activity that can be used for the development of a natural herbicide [10,19]. In the framework of our ongoing research on phytotoxic ten-membered lactones, we report here the results of SAR analysis for these compounds.

Despite the great diversity of the known ten-membered lactones, little research efforts were devoted to their SAR [12,16,20,21]. Having analyzed the phytotoxic activities within stagonolides, herbarumins and putaminoxins, Evidente et al. (2008) concluded that modification at the C-2−C-4 site, as well as the replacement of the propyl substituent at the C-9 with methyl group, leads to a loss of phytotoxic activity [16]. The major ten-membered lactones of *S. cirsii* have a similar skeleton with non-substituted C-2–C-4 sites but differ by the presence and configuration of hydroxy groups at C-7−C-8 and by the configuration of the propyl chain at C-9. The aim of this study was to create a set of structurally related ten-membered lactones and to reveal the structural features that affect their biological activity.

## 2. Materials and Methods

### 2.1. General Experimental Procedures

The ^1^H and ^13^C NMR spectra were recorded at 400 and at 100 MHz, respectively, in CDCl_3_ as a solvent on a Bruker AVANCE III 400 MHz spectrometer (Bruker, Karlshrue, Germany). The solvent residual signal (δ 7.26 ppm) for ^1^H NMR spectra and the carbon signal of CDCl_3_ (δ 77.16 ppm) for ^13^C NMR spectra were used as references. Distortionless enhancement by polarization transfer (DEPT), correlation spectroscopy (COSY)-45, total correlation spectroscopy (TOCSY), nuclear Overhauser effect spectroscopy (NOESY), heteronuclear multiple-quantum coherence (HSQC) and heteronuclear multiple-bond correlation (HMBC) were performed using standard Bruker microprograms. ESI-MS spectra were recorded with TSQ Quantum Access spectrometer (Thermo Scientific, Waltham, MA, USA) after HPLC. Analytical TLC was performed on silica gel Kieselgel 60 F_254_ plates (Merck, Darmstadt, Germany). The spots were visualized by exposure to UV radiation (254 nm) and/or by spraying with reagent anisaldehyde–H_2_SO_4_, followed by heating at 120 ℃ for 2 min. Medium-pressure chromatography (MPLC) was performed with a Sepacore chromatography system (Büchi, Flawil, Switzerland) using prepackaged normal-phase (Silica HP 50 μm 120 g and Silica HP 30 μm 40 g) Puriflash columns (Interchim, Montluçon, France). The following chromatographic system was used for preparative HPLC: Quaternary Gradient Module 2545, UV/Visible Detector 2489 and Fraction Collector III (Waters, Milford, MA, USA). Dichloromethane for synthesis was dried over calcium chloride and distilled over phosphorus pentoxide. Pyridine was dried over sodium hydroxide and distilled over sodium slices. Barium manganate was obtained according to previously described procedure [22]. The rest of the starting materials were obtained commercially and used without further purification.

### 2.2. Fungal Strain and Toxin Production

The strain S-47 of *Stagonospora cirsii* Davis used in this study has been deposited in the collection of the All-Russian Institute of Plant Protection as VIZR G-51 (Pushkin, Saint Petersburg, Russia). The submerged fermentation was conducted in a 7 L fermenter (Applikon Biotechnology, Delft, The Netherlands) containing 5 L of modified Czapek medium (g/L of deionized water, pH 6: glucose—45, NaNO**_3_**—3, KH**_2_**PO**_4_**—1, MgSO**_4_** × 7H**_2_**O—0.5, KCl—0.5, FeSO**_4_** × 7H**_2_**O—0.01, CaCl**_2_** × 2H**_2_**O—1 × 10**^−4^**, H**_3_**BO**_3_**—0.01, ZnSO**_4_** × 7H**_2_**O—0.004, thiamine—1 × 10**^−4^**, biotin—5 × 10**^−6^**). The fermentation of the fungus, as well as extraction and purification of stagonolides A (**1**), J (**2**), K (**3**) and herbarumin I (**4**), were performed as described previously [10,18].

### 2.3. Semisynthetic Derivatives Preparation

Compound **5**—8-acetylherbarumin I ((5E,7S,8S,9R)-7-hydroxy-8-acetoxy-9-propyl-5-nonen-9-olide)

To a solution of herbarumin I (**4**) (60 mg, 0.263 mmol) in dry pyridine (0.6 mL) acetic anhydride (49 μL, 0.526 mmol) was added. The mixture was stirred at room temperature for 20 h. After completion of the reaction, the mixture was diluted with hexane (30 mL) and washed with 10% aqueous HCl solution (10 mL). The aqueous layer was extracted with hexane (30 mL). The combined organic layer was washed with distilled water (2 × 30 mL), dried with anhydrous Na_2_SO_4_, filtered and the filtrate was evaporated in vacuo. The crude product was purified using HPLC (XBridge Prep C18 5μm, column size 10 mm × 250 mm, elution with 65% acetonitrile in 0.1% formic acid, flow rate 6.6 mL/min, detection 190 nm and 225 nm, t_R_ 3.0 min). The product **5** was obtained as colorless oil (33.2 mg, 47% yield), MS (ESI) (+) m/z 271 [M+H]^+^, 253 [M+H-H_2_O]^+^, 211 [M+H-H_2_O-AcOH]^+^, 193 [M+H-2H_2_O-AcOH]^+^; ^1^H NMR (400 MHz, CDCl_3_): δH 0.91 (t, 3H, *J* 7.4 Hz, CH_3_-12), 1.22–1.42 (m, 2H, CH_2_-11), 1.43–1.59 (m, 2H, CH_2_-10), 1.71–1.80 (m, 1H, H-3a), 1.84–2.09 (m, 3H, H-3b, H-4a, H-2a), 2.15 (s, 3H, CH_3_-14), 2.35 (ddd, 1H, *J* 2.1, 5.6, 13.8 Hz, H-2b), 2.39–2.47 (m, 1H, H-4b), 4.45 (bs, 1H, H-7), 4.87 (dd, 1H, *J* 2.1, 10.2 Hz, H-8), 5.33 (td, 1H, *J* 9.5, 3.4 Hz, H-9), 5.54–5.67 (m, 2H, H-5, H-6); ^13^C NMR (100 MHz, CDCl_3_): δC 13.8 (C-12), 17.7 (C-11), 21.1 (C-14), 24.5 (C-3), 33.2 (C-10), 33.4 (C-4), 34.4 (C-2), 67.6 (C-9), 71.1 (C-7), 74.7 (C-8), 125.7 (C-5), 130.1 (C-6), 169.9 (C-13), 175.9 (C-1).

Compound **6**—7,8-bis(acetyl)herbarumin I ((5E,7S,8S,9R)-7,8-diacetoxy-9-propyl-5-nonen-9-olide)

To a solution of **4** (100 mg, 0.438 mmol) in dry pyridine (2 mL) acetic anhydride (2.50 mL, 26.496 mmol) was added. The mixture was stirred at room temperature for 19 h. After completion of the reaction, the mixture was poured into cold 10% aqueous HCl solution (30 mL). The aqueous layer was extracted with hexane (3 × 25 mL). The combined organic layer was washed with distilled water (2 × 50 mL), dried with Na_2_SO_4_, filtered and the filtrate was evaporated in vacuo. The product **6** was obtained as a white solid (133.2 mg, 97% yield); MS (ESI) (+) m/z 313 [M+H]^+^, 253 [M+H-H_2_O-AcOH]^+^, 211 [M+H-H_2_O-2AcOH]^+^, 193 [M+H-2H_2_O-2AcOH]^+^; ^1^H NMR (400 MHz, CDCl_3_): δH 0.91 (t, 3H, *J* 7.2 Hz, CH_3_-12), 1.25–1.47 (m, 3H, CH_2_-11, H-10a), 1.49–1.60 (m, 1H, H-10b), 1.71–1.79 (m, 1H, H-3a), 1.81–2.00 (m, 2H, H-4a, H-3b), 2.01–2.10 (m, 1H, H-2a), 2.05 (s, 3H, CH_3_-16), 2.19 (s, 3H, CH_3_-14), 2.32–2.42 (m, 2H, H-2b, H-4b), 4.92 (dd, 1H, *J* 2.4, 10.1 Hz, H-8), 5.34–5.47 (m, 2H, H-5, H-9), 5.59 (dd, 1H, *J* 15.6, 1.2 Hz, H-6), 5.65–5.71 (m, 1H, H-7); ^13^C NMR (100 MHz, CDCl_3_): δC 13.9 (C-12), 17.5 (C-11), 20.8 (C-16), 20.9 (C-14), 24.4 (C-3), 33.4 (C-4, C-10), 34.5 (C-2), 68.5 (C-9), 71.0 (C-7), 72.3 (C-8), 125.3 (C-5), 126.9 (C-6), 169.7 (C-15), 170.1 (C-13), 174.8 (C-1). Additionally, the product **6** was obtained as a by-product in the preparation of compound **5** (white solid, 9.8 mg, 12% yield, t_R_ 4.2 min).

Compound **7**—7-acetylstagonolide J ((5E,7R*,8S*,9R*)-7-acetoxy-8-hydroxy-9-propyl-5-nonen-9-olide)

To a solution of stagonolide J (**2**) (58 mg, 0.254 mmol) in dry pyridine (1 mL) acetic anhydride (36 μL, 0.381 mmol) was added. The mixture was stirred at room temperature for 22 h. After completion of the reaction, the mixture was diluted with hexane (30 mL) and washed with 10% aqueous HCl solution (10 mL). The aqueous layer was extracted with hexane (10 mL). The combined organic layer was washed with distilled water (2 × 30 mL), dried with Na_2_SO_4_, filtered and the filtrate was evaporated in vacuo. The crude products were purified using HPLC (XBridge Prep C18 5 μm, column size 10 mm × 250 mm, elution with 55% acetonitrile in 0.1% formic acid, flow rate 6.6 mL/min, detection 190 nm and 225 nm, t_R_ 3.8 min). The product **7** was obtained as a white solid (12.9 mg, 19% yield); MS (ESI) (+) m/z 271 [M+H]^+^, 253 [M+H-H_2_O]^+^, 211 [M+H-H_2_O-AcOH]^+^; ^1^H NMR (400 MHz, CDCl_3_): δH 0.94 (t, 3H, *J* 7.4 Hz, CH_3_-12), 1.25–1.45 (m, 2H, CH_2_-11), 1.51–1.62 (m, 1H, H-10a), 1.71–1.84 (m, 1H, H-3a), 1.87–1.97 (m, 3H, H-3b, H-4a, H-10b), 1.99–2.08 (m, 1H, H-2a), 2.09 (s, 3H, CH_3_-14), 2.27 (d, 1H, *J* 4.7 Hz, OH), 2.32–2.43 (m, 2H, H-2b, H-4b), 3.60 (td, 1H, *J* 4.6, 9.4 Hz, H-8), 4.98 (td, 1H, *J* 2.7, 9.3 Hz, H-9), 5.05 (t, 1H, *J* 9.5 Hz, H-7), 5.43 (ddd, 1H, *J* 9.7, 15.4, 0.6 Hz, H-6), 5.64–5.74 (m, 1H, H-5); ^13^C NMR (100 MHz, CDCl_3_): δC 13.9 (C-12), 17.7 (C-11), 21.3 (C-14), 24.4 (C-3), 33.4 (C-4), 33.7 (C-10), 34.4 (C-2), 72.8 (C-9), 73.1 (C-8), 79.3 (C-7), 128.4 (C-6), 134.2 (C-5), 170.6 (C-13), 175.0 (C-1).

Compound **8**—7,8-bis(acetyl)stagonolide J ((5E,7R*,8S*,9R*)-7,8-diacetoxy-9-propyl-5-nonen-9-olide)

To a solution of **2** (18.7 mg, 0.082 mmol) in dry pyridine (0.3 mL) acetic anhydride (155 μL, 1.639 mmol) was added. The mixture was stirred at room temperature for 23 h. After completion of the reaction, the mixture was poured into cold 10% aqueous HCl solution (10 mL). The aqueous layer was extracted with hexane (2 × 10 mL). The combined organic layer was washed with distilled water (2 × 30 mL), dried with anhydrous Na_2_SO_4_, filtered and the filtrate was evaporated in vacuo. The product **8** was obtained as a white solid (21.2 mg, 83% yield); MS (ESI) (+) m/z 253 [M+H-H_2_O-AcOH]^+^, 211 [M+H-H_2_O-2AcOH]^+^, 193 [M+H-2H_2_O-2AcOH]^+^; ^1^H NMR (400 MHz, CDCl_3_): δH 0.90 (t, 3H, *J* 7.3 Hz, CH_3_-12), 1.19–1.41 (m, 2H, CH_2_-11), 1.41–1.57 (m, 2H, CH_2_-10), 1.72–1.84 (m, 1H, H-3a), 1.85–2.02 (m, 2H, H-3b, H-4a), 1.99 (s, 3H, CH_3_-14), 2.02–2.12 (m, 1H, H-2a), 2.07 (s, 3H, CH_3_-16), 2.32–2.44 (m, 2H, H-2b, H-4b), 5.03–5.13 (m, 2H, H-8, H-9), 5.17–5.28 (m, 1H, H-7), 5.48 (dd, 1H, *J* 9.7, 15.4 Hz, H-6), 5.63–5.76 (m, 1H, H-5); ^13^C NMR (100 MHz, CDCl_3_): δC 13.8 (C-12), 17.7 (C-11), 20.8 (C-16), 21.0 (C-14), 24.3 (C-3), 33.3 (C-10, C-4), 34.2 (C-2), 70.9 (C-9), 72.2 (C-8), 76.0 (C-7), 128.0 (C-6), 134.4 (C-5), 169.6 (C-13), 169.7 (C-15), 174.9 (C-1). Additionally, the product **8** was obtained as a by-product in the preparation of compound **7** (white solid, 13.0 mg, 16% yield, t_R_ 6.3 min).

Compound **9**—Acetylstagonolide K ((5E,7R,9S)-7-acetoxy-9-propyl-5-nonen-9-olide)

To a solution of stagonolide K (**3**) (57 mg, 0.269 mmol) in dry pyridine (1 mL) acetic anhydride (0.51 mL, 5.374 mmol) was added. The mixture was stirred at room temperature for 19 h. After completion of the reaction, the mixture was poured into cold 10% aqueous HCl solution (15 mL). The aqueous layer was extracted with hexane (3 × 15 mL). The combined organic layer was washed with distilled water (2 × 30 mL), dried with Na_2_SO_4_, filtered and the filtrate was evaporated in vacuo. The product **9** was obtained as colorless oil (61.8 mg, 91% yield); MS (ESI) (+) m/z 195 [M+H-H_2_O-AcOH]^+^; ^1^H NMR (400 MHz, CDCl_3_): δH 0.89 (t, 3H, *J* 7.3 Hz, CH_3_-12), 1.23–1.38 (m, 2H, CH_2_-11), 1.38–1.58 (m, 2H, CH_2_-10), 1.71–1.84 (m, 2H, H-3a, H-8a), 1.84–1.90 (m, 1H, H-8b), 1.90–2.07 (m, 3H, H-2a, H-3b, H-4a), 1.99 (s, 3H, CH_3_-14), 2.25–2.38 (m, 2H, H-2b, H-4b), 5.04–5.18 (m, 2H, H-7, H-9), 5.42 (ddd, 1H, *J* 9.5, 15.4, 0.8 Hz H-6), 5.54–5.65 (m, 1H, H-5); ^13^C NMR (100 MHz, CDCl_3_): δC 13.8 (C-12), 18.2 (C-11), 21.3 (C-14), 26.1 (C-3), 33.5 (C-4), 34.6 (C-2), 37.7 (C-10), 38.7 (C-8), 70.6 (C-9), 74.5 (C-7), 131.5 (C-6), 133.3 (C-5), 169.9 (C-13), 175.3 (C-1).

Compound **10**—Acetylstagonolide A ((5E,8S,9R)-7-oxo-8-acetoxy-9-propyl-5-nonen-9-olide)

To a solution of **5** (33 mg, 0.123 mmol) in dry dichloromethane (3 mL) manganese dioxide (214 mg, 2.456 mmol) was added. The mixture was stirred at room temperature for 40 h then filtered through a syringe filter (CHROMAFIL^®^ Xtra PA, 25 mm, 0.45 μm) and evaporated in vacuo. The crude product was purified using HPLC (XBridge Prep C18 5 μm, column size 10 mm × 250 mm, elution with 40% acetonitrile in 0.1% formic acid, flow rate 6.6 mL/min, detection 190 nm and 225 nm, t_R_ 8.6 min). The product **10** was obtained as a colorless oil (13.9 mg, 42% yield); MS (ESI) (+) m/z 269 [M+H]^+^, 251 [M+H-H_2_O]^+^, 209 [M+H-H_2_O-AcOH]^+^, 191 [M+H-2H_2_O-AcOH]^+^; ^1^H NMR (400 MHz, CDCl_3_): δH 0.94 (t, 3H, *J* 7.4 Hz, CH_3_-12), 1.28–1.48 (m, 2H, CH_2_-11), 1.55–1.67 (m, 1H, H-10a), 1.72–1.84 (m, 1H, H-10b), 1.88–2.09 (m, 3H, H-3a, H-3b, H-4a), 2.12–2.23 (m, 1H, H-2a), 2.19 (s, 3H, CH_3_-14), 2.43–2.57 (m, 2H, H-2b, H-4b), 4.94 (d, 1H, *J* 10.3 Hz, H-8), 5.10 (td, 1H, *J* 9.8, 2.6 Hz, H-9), 6.20–6.31 (m, 1H, H-5), 6.47 (d, 1H, *J* 15.9 Hz, H-6); ^13^C NMR (100 MHz, CDCl_3_): δC 13.7 (C-12), 17.8 (C-11), 20.5 (C-14), 24.7 (C-3), 33.5 (C-4), 34.0 (C-2, C-10), 71.5 (C-9), 77.4 (C-8), 132.4 (C-6), 143.1 (C-5), 169.6 (C-13), 173.9 (C-1), 194.0 (C-7).

Compound **11**—C-7 oxidized stagonolide K ((5E,9S)-7-oxo-9-propyl-5-nonen-9-olide)

To a solution of **3** (48 mg, 0.226 mmol) in dry dichloromethane (6 mL) barium manganate (579 mg, 2.263 mmol) was added. The mixture was stirred at room temperature for 48 h then filtered through a syringe filter (CHROMAFIL^®^ Xtra PA, 25 mm, 0.45 μm) and evaporated in vacuo. The crude product was purified using HPLC (XBridge Prep C18 5 μm, column size 10 mm × 250 mm, elution with 40% acetonitrile in 0.1% formic acid, flow rate 6.6 mL/min, detection 190 and 225 nm, t_R_ 7.0 min). The product **11** was obtained as a colorless oil (8.6 mg, 18% yield); MS (ESI) (+) m/z 211 [M+H]^+^, 193 [M+H-H_2_O]^+^; ^1^H NMR (400 MHz, CDCl_3_): δH 0.93 (t, 3H, *J* 7.3 Hz, CH_3_-12), 1.28–1.47 (m, 2H, CH_2_-11), 1.48–1.72 (m, 2H, CH_2_-10), 1.90–2.00 (m, 1H, H-3a), 2.01–2.20 (m, 3H, H-2a, H-3b, H-4a), 2.38–2.61 (m, 3H, H-2b, H-4b, H-8a), 2.73 (dd, 1H, *J* 11.9 Hz, *J* 2.6 Hz, H-8b), 5.10–5.21 (m, 1H, H-9), 6.21 (ddd, 1H, *J* 2.6, 10.6, 15.8 Hz, H-5), 6.35 (d, 1H, *J* 16.1 Hz, H-6); ^13^C NMR (100 MHz, CDCl_3_): δC 13.6 (C-12), 18.5 (C-11), 26.0 (C-3), 33.5 (C-4), 34.3 (C-2), 36.7 (C-10), 47.0 (C-8), 71.6 (C-9), 135.3 (C-6), 142.9 (C-5), 174.8 (C-1), 199.7 (C-7).

The NMR spectra and ESIMS of semisynthetic derivatives **5**–**11** are given in the Supplementary Information.

### 2.4. Biological Activity Assays

All compounds were tested after HPLC purification; the purity was 96–98% (NMR controlled). Two herbicides belonging to different classes were used as positive controls in phytotoxicity bioassays—metribuzin (triazine class) (Sigma-Aldrich, St. Louis, MO, USA) and metsulfuron-methyl (sulfonylurea class) (Dr. Ehrenstorfer, Augsburg, Germany).

#### 2.4.1. Leaf Puncture Assay

The samples of compounds to be assayed (0.3 mg each) were dissolved in 7.5 μL of EtOH and adjusted to the volume of 150 μL with water. The final concentration of ethanol was 5% (*v*/*v*), the concentration of the tested toxins was 2 mg/mL, 5% EtOH was used as control treatment. Punctured leaf discs of perennial sowthistle (*Sonchus arvensis*) were placed in a wet chamber and treated with 10 μL of test solutions [19,23]. The diameter of the necrotic lesions was measured 48 and 120 h after treatment. Twelve leaf discs (replicates) were used for each treatment.

#### 2.4.2. Seedlings Bioassay

Water agar was prepared by autoclaving 3 g Bactoagar (BD Difco, Fisher Scientific, Göteborg Sweden) in 400 mL distilled water for 20 min. Then, 1 mL of molten agar was poured in each well of the 24-well plate. Lettuce (*Lactuca sativa*) seeds were surface-sterilized in commercial bleach for 1 min, thoroughly rinsed with sterile distilled water and imbibed for 4 h on two layers of filter paper that were saturated with water. A stock solution of each test compound was prepared by dissolving 8 mg in 20 μL chloroform and diluting to 400 μL with hexane. A dosage series of each compound was prepared by diluting the stock solution with 5:95 chloroform–hexane (*v*/*v*) to concentrations ranging from 0.5 to 20.0 mg/mL. Next, 50 μL of each solution were carefully layered on the solidified agar in each well, so that the doses of test-compounds ranged from 25 to 1000 μg per well (or from 5 to 200 μg/seed). The solvent was allowed to passively evaporate from the agar surface in a ventilated hood, typically requiring 30 min before seeds were placed on the agar. Five seeds were placed on the agar in each well. The plates were incubated in a growth chamber at constant 24 °C. After three days of incubation, the length (root plus shoot) of the seedlings was measured after being frozen [24]. Fifteen seedlings (three replications of five seedlings each) were used for each treatment, and two independent experiments were performed.

#### 2.4.3. Microalgae Assay

*Haematococcus lacustris* Flotow strain IMBR-1 “Adler” was isolated from subtropical valleys of the Black Sea region [25]. Cells of *H. lacustris* were propagated photoautotrophically in a 1000 mL Erlenmeyer flask containing 500 mL liquid Optimized Haematococcus Medium (OHM) [26]. OHM contained KNO_3_ (410 mg/L), Na_2_HPO_4_ (30 mg/L), MgSO_4_ × 7H_2_O (246.5 mg/L), CaCl_2_ (48.1 mg/L), FeC_6_H_5_O_7_ × 5H_2_O (2.62 mg/L), CoCl_2_ × 6H_2_O (0.011 mg/L), CuSO_4_ × 5H_2_O (1.012 mg/L), Cr_2_O_3_ (0.076 mg/L), MnCl_2_ × 4H_2_O (0.989 mg/L), Na_2_MoO_4_ × 2H_2_O (0.12 mg/L), Na_2_SeO_2_ (0.008 mg/L), ZnSO_4_ × 7H_2_O (0.1 mg/L) and thiamine (17.5 mg/L). Prior to use, OHM was sterilized at 121 °C for 20 min. Cells were grown at 24 °C under illumination with cool-white fluorescent tubes at a continuous light intensity with a PPFD (400–700 nm) of 25 μmol m-2s-1. For experiments, 30 mL aliquots of the algal suspension were transferred to sterile 50 mL Erlenmeyer flasks. Algae cultures were incubated for 24 h before the addition of the toxins. The initial cell concentration was 5×10^4^ cells/mL. A stock solution of each test compound was prepared by dissolving 5 mg in 100 μL of dimethyl sulfoxide (DMSO). A dosage series of each compound was prepared by diluting the stock solution with DMSO to concentrations ranging from 1 to 50 mg/mL. *H. lacustris* cultures were then supplemented with 30 μL of toxin solutions per flask. The final concentration of DMSO was 0.01% (*v*/*v*); the concentration of the tested toxins was in the range of 1 to 50 µg/mL (*w*/*v*) while 0.01% DMSO was used as control treatment. Microalgae cultures were incubated for 48 h at 24 °C and a continuous light intensity of 25 μmol m-2s-1. The viability of algal cells was determined via staining with Evans Blue dye [27]. Aliquots (1000 μL) of toxin-treated and control cell cultures, respectively, were pipetted into Eppendorf tubes; then, 100 μL of Evans Blue (0.5% in water) were added and the tubes were incubated for 15 min. Cells were centrifuged for 5 min at 600× *g*, washed three times with OHM and resuspended in 100 μL of OHM. The obtained cell suspensions were analyzed using Olympus BX51 microscope (Olympus Deutschland GmbH, Hamburg, Germany) equipped with a ColorView II digital camera and Cell ^F image analytical software (V1.11, Olympus Soft Imaging Solutions, Münster, Germany). Fifty cells in three fields per toxin/concentration were analyzed.

#### 2.4.4. Antimicrobial Assay

The antimicrobial activity of the compounds **1**–**11** was tested against *Bacillus subtilis* NCTC 104000 by using the paper-disc agar diffusion assay [28]. Bacteria was grown on potato dextrose agar. The samples of assayed compounds were dissolved in acetone and applied to the 6 mm paper discs (Macherey-Nagel, Düren, Germany) at a concentration 100 µg/disc. The treated microbial cultures were incubated at 30 °C for 24 h before activity was determined as the radius of the growth inhibition zone in mm.

#### 2.4.5. Cytotoxicity Assay

The cytotoxic activity of the compounds **1**–**11** was studied with the Sf9 cell line (ECACC 89070101) of the fall armyworm (*Spodoptera frugiperda*) maintained at the Laboratory of Microbiological Plant Protection (VIZR, St. Petersburg, Russia). Samples of assayed compounds were dissolved in DMSO to concentration 0.1–10.0 mg/mL. 10 μL of toxin solutions were added to the wells of a 48-well plate, then 890 μL of SF900II culture medium (Thermo Fisher Scientific, Waltham, MA, USA) and 100 μL of a suspension of actively growing cells (viability not lower than 90%) were added to each well at a concentration of 3 × 10^5^ cells/well. As a result, the concentrations of the tested compounds were 1–100 μg/mL, and the solvent concentration was 1%. Additionally, 10 μL of DMSO was used as a control treatment. The cells were incubated for 24 h at 27 °C and stained with trypan blue, and the percentage of dead (stained) cells was determined in relation to the total number (at least 50) in several fields of view [29].

### 2.5. LogP determination

Determination of logP was conducted using a reversed-phase HPLC method. HPLC analysis of samples and six standards was carried out using TSQ Quantum AccessTM (Thermo Fisher Scientific, Waltham, MA, USA) chromatographic system equipped with column Zorbax SB-C18 (Agilent Tech., Santa Clara, CA, USA, pore size 1.8 μm, 4.6 × 150 I.D.). Each analysis was performed isocratically using a mixture acetonitrile–0.1% formic acid 40:60 (*v*/*v*) as an eluent with flow rate 1 mL/min. Six active ingredients of pesticides of known logP were used as standards in the determination of logP values [30,31]. The retention factors (k′) were calculated according to the equation k′ = (t_R_ − t_M_)/t_M_, where t_R_–retention time of standard, min (Table 1); t_M_—the holdup time of the system (1.53 min). The calibration line for the RP-HPLC retention factors was established by linear regression with the reference data: logP = 1.7709 × logk′ + 0.9866 (*r*^2^ = 0.986).

### 2.6. Statistical Analysis

The data of bioassays were subjected to one-way ANOVA performed using Statistica 8.0 (StatSoft, Tusla, OK, USA); the differences between toxin treatments were considered significant at *p* < 0.05. The ID_50_ values representing the toxin doses required to cause a 50% reduction in seedlings length compared to control treatment, and LC_50_ values representing the toxin concentration required to cause a 50% reduction in microalgae and Sf9 cells viability, were determined by the curvilinear regression procedure of Sigma Plot 14.0 (Systat Software, San Jose, CA, USA). If not indicated otherwise, three independent experiments were performed per assay/treatment.

Biological data and logP were subjected to principal component analysis (PCA) (Sigma Plot 14.0) to the discrimination of compounds **1**–**11**. The data for PCA were used as they appear in Table 2 except for the data of seedlings bioassay which were expressed as inhibition of seedlings growth compared to control (%) at a dose 40 μg/seed. All data were standardized prior to the analysis.

## 3. Results

**Figure 2:**
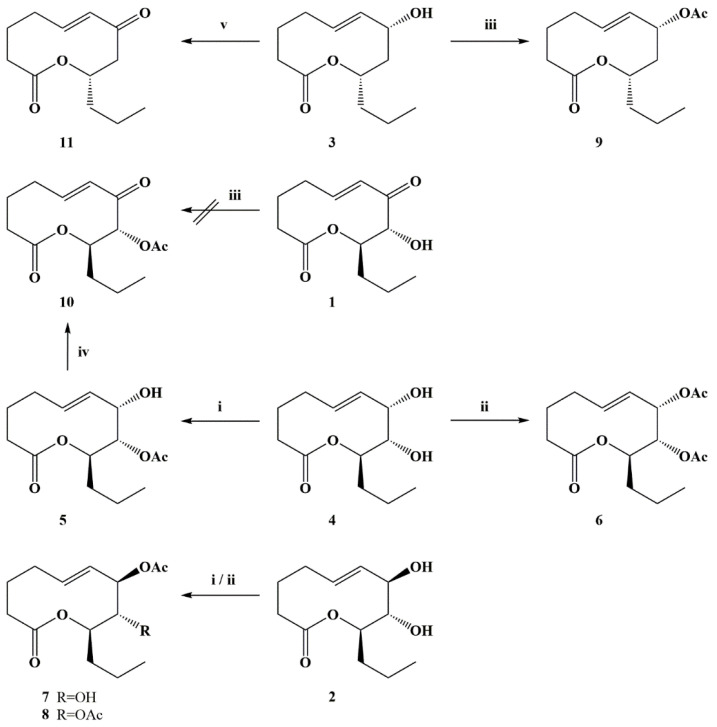
Scheme of modification of natural nonenolides **1—4**. *Reagents and conditions:* (i) 2 eq Ac_2_O, Py, r.t.; (ii) 60 eq Ac_2_O, Py, r.t.; (iii) 20 eq Ac_2_O, Py, r.t.; (iv) 20 eq MnO_2_, CH_2_Cl_2_, r.t.; (v) 10 eq BaMnO_4_, CH_2_Cl_2_, r.t.

In order to better understand the structure–activity relationships of phytotoxic ten-membered lactones, isolated from *Stagonospora cirsii* G-51 VIZR (stagonolides A (**1**), J (**2**), K (**3**) and herbarumin I (**4**)), some of their semisynthetic derivatives (**5**–**11**) were prepared (Figure 1, Figure 2). Esters (**5**–**9**) were prepared by acetylation of compounds **2**–**4** using acetic anhydride in pyridine solution. Using two-fold excess of acylating reagent, herbarumin I (**4**) afforded a mixture of 8-monoacetyl- (**5**) and 7,8-bis(acetyl)derivatives (**6**) with 47 and 12% yields, respectively. Stagonolide J (**2**) demonstrated regioselectivity different from its C-7 epimer herbarumin I (**4**). Acetylation of **2** using 1.5-fold excess of acetic anhydride results in the mixture of mono- (**7**) and bis-acetylated (**8**) derivatives isolated with 19 and 16% yields, respectively. However, in this case acetyl moiety is located at the C-7 position of monoacetate (**7**) as it was deduced from HMBC and NOESY spectra analysis. However, during this reaction, traces of 8-monoacetyl derivative were observed as an impurity. Bis(acetyl)nonenolides **6** and **8** were selectively obtained with 97 and 83% yields, respectively, using a large excess of acetic anhydride. Stagonolide K (**3**) acetylation was also well performed using a large excess of acylating reagent with 91% yield (Figure 2). An attempt to synthesize the compound **10** by the same method led to the total loss of starting material while no desired product was detected by TLC. Nevertheless 8-acetylstagonolide A (**10**) was prepared by mild oxidation of 8-acetylherbarumin I (**5**) using manganese dioxide with 42% yield (Figure 2). C-7 oxidation of **3** using 20-fold excess of manganese dioxide or 10-fold excess of barium manganate afforded the product **11** with low yields 13–18%. Though the reaction was performed selectively, almost half of the starting material was recovered using HPLC-purification of the mixture (Figure 2).

In leaf puncture bioassay stagonolide A (**1**) and C-7 oxidized stagonolide K (**11**) showed the highest phytotoxic activity (the average diameters of necrotic lesions were 6.4 and 5.2 mm, respectively). Neither 7-acetylstagonolide J (**7**) or bis(acetyl)stagonolide J (**8**) displayed any toxicity in this bioassay. Other compounds showed moderate activity. Notably, the response of sowthistle leaves to **1**, **10** and **11** was observed already several hours after the treatment and was seen as wide necrotic lesions, while the maximal effect of **2** and **4** developed only to the fifth day after the treatment (Table 2; Figure S48 in Supplementary Information). Metribuzin caused the formation of necrotic lesions with the average diameter 5.4 mm at 120 h after the treatment, while metsulfuron-methyl was inactive in this bioassay (Table 2).

The sensitivity of lettuce seedlings to the ten-membered lactones **1**–**11** differed from that of punctured sowthistle leaves. Compounds **1** and **11** were the most toxic to the seedlings (ID_50_ of 0.03 and 0.04 μmole/seed, respectively). Herbarumin I (**4**) was less phytotoxic in this bioassay (ID_50_ of 0.35 μmole/seed), while its mono- (**5**) and bis(acetyl)- (**6**) derivatives inhibited the seedlings growth with ID_50_ 0.04 and 0.07 μmole/seed, respectively. Stagonolide J (**2**) and its acetyl derivatives **7** and **8** did not affect the lettuce seedlings at a dose up to 80 × 10^−8^ mole/seed (Table 2). The activity levels of **1**, **5**, **6** and **11** were comparable to that of metsulfuron-methyl with ID_50_ of 0.03 μmole/seed, while metribuzin was less toxic in this bioassay.

Among 11 nonenolides tested, only **1**, **10** and **11** displayed acute toxicity to *H. lacustris* and Sf9 cells in micromolar concentrations and also displayed antimicrobial activity to *Bacillus subtilis* (Table 2).

The octanol/water partition coefficient (logP) was used as a lipophilicity characteristic (Table 2). Among tested compounds acetyl derivatives **6**, **8** and **9** were the most lipophilic ones. Acetylated structural analogues **5** and **7**, as well as **6** and **8** had similar logP values (2.32; 2.21 and 3.18; 3.20, respectively), that are higher than those of their natural precursors **2** and **4** (Table 2). 

**Figure 3:**
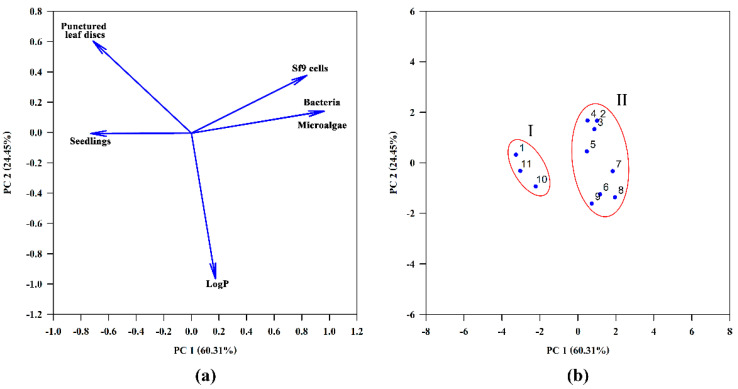
Principal component analysis (PCA) among biological activity and logP of the compounds **1**–**11**: (**a**) loading plot, (**b**) score plot. The red circles indicate the separated groups (subsets) of the studied compounds.

The activity data and logP of compounds **1**–**11** were subjected to Principal Component Analysis (PCA) for a better visualization of the data set (Figure 3). The data obtained by different bioassays have been standardized prior to the analysis. The PC1 and PC2 axes corresponded to 60.31 and 24.45% of the total variance of the original data, respectively. While the first component, PC1, indicates that dissimilarities across the horizontal axis are mostly due to the distinct biological activities, the second component, PC2, reflects the contribution of logP to the differentiation of compounds. The loading plot shows that toxicity of compounds to bacteria, green microalgae and Sf9 cells are highly correlated, whereas logP demonstrated no correlation with seedlings toxicity and negative correlation with leaf-puncture assay data (Figure 3A). The compounds have been separated into two subsets I and II, horizontally opposed, indicating that PC1 was the major factor responsible for this characterization (Figure 3B). Subset I includes stagonolide A (**1**), its acetyl derivative (**10**) and С-7 oxidized stagonolide K (**11**) demonstrated biological activity in all used bioassays (Table 2). The common structural feature of these compounds that apparently determines their general toxicity is the presence of a carbonyl group at C-7. The similar response profile of compounds **1**, **10** and **11** indicated that the configuration of the propyl chain at C-9 (*R* in **1** and **10**, *S* in **11**) (Figure 1) is not important for biological activity. The acetylation of the hydroxyl group at C-8 in **10** or its absence in **11** (Figure 2) resulted in decrease in activity in all employed bioassays compared to **1**. The compounds that do not possess the carbonyl group in their structure (**2**–**9**) are characterized by the lack of toxicity to microalgae and bacteria and form the subset II (Figure 3). Compounds **2**–**8** were also non-toxic to Sf9 cells whereas the acetylation of stagonolide K resulted in the manifestation of weak cytotoxic activity of **9** to Sf9 cells (Table 2). Stagonolide J (**2**) was toxic only to punctured leaf discs of sowthistle whereas its mono- (**7**) and bis-acetyl (**8**) derivatives totally lost phytotoxic activity. Therefore, the natural and semisynthetic ten-membered lactones with *R* configuration of C-7 (**2**, **7**, **8**) were much less phytotoxic than their 7*S* analogues (**4**, **5**, **6**, respectively).

## 4. Discussion

The structure–activity relationship (SAR) of many potent natural products (sphaeropsidins [33], radicinins and radicinols [34], fumonisins [35] and others) was analyzed using semisynthesis to expand the compound libraries. However, in previous SAR studies of ten-membered lactones (nonenolides) a few semisynthetic derivatives were included into the libraries due to the low yield of these natural products from producing fungal cultures. The biotechnological production of nonenolides using different cultures of *Stagonospora cirsii* [18] opens up the possibility of their chemical modification. The structural diversity and high yield of *S. cirsii* ten-membered lactones allowed us to create a set of eleven compounds for SAR analysis using simple chemical reactions for the structural modification. However, these natural products turned out to be quite challenging molecules for semisynthesis due to the insufficient data about their reactivity. A few publications described the preparation of derivatives of natural nonenolides, however, most of these works were aimed at proving the elucidated structure. This approach was used by Evidente et al. to confirm the structures of pinolidoxin from *Ascochyta pinodes* [36], putaminoxin A [14] and D [20], the phytotoxins of *Phoma putaminum*, and others. The typical employed reactions included acetylation by the reaction with acetic anhydride in pyridine and catalytic hydrogenation on presaturated PtO_2_. In our research, the acetylation of ten-membered lactones **2**–**4** worked well when no regioselectivity was needed. Apparently, steric effects play a major role in reactivity of hydroxyl moieties of vicinal diols (**2**, **4**). Thus, differences in C-7 configuration significantly affected the structure of monoacetates (**5**, **7**). Among nonenolides **1**–**4** only stagonolide A (**1**) demonstrated a negative response to acetic anhydride and pyridine. Unexpected loss of stability of **1** under such mild conditions will be a subject for further research. However, the lability of stagonolide A (**1**) in this case is not an obstacle as acetylstagonolide A (**10**) could be prepared from herbarumin I (**4**) by two step synthesis (Figure 2).

A created set of ten-membered lactones **1**–**11** were studied in terms of their target (phytotoxic) and non-target (cytotoxic, antimicrobial) biological activities. Previous studies of stagonolide A, herbarumin I and related compounds primarily concerned their phytotoxic activity estimated with leaf puncture assay and seedlings assay. Stagonolide A (**1**) demonstrated phytotoxic activity on punctured leaves of sowthistle [10] and Canada thistle [15] as well as against Canada thistle seedlings [15]. Herbarumin I (**4**) inhibited the growth of *Amaranthus hypochondriacus* seedlings [4] and caused necrotic lesions on sowthistle punctured leaves [10]. In our previous studies stagonolide A (**1**) and herbarumin I (**4**) demonstrated the potent post-emergent herbicidal activity against perennial sowthistle aerial shoots, the treatment of plants with these toxins supplemented with Hasten™ led to the development of wide necrotic lesions on the leaf surface [19]. For adequate assessment of future perspectives of most phytotoxic compounds it is important to compare their target activity with that of chemical herbicides. Metribuzin and metsulfuron-methyl are widely used herbicides that have different modes of action (inhibitors of photosystem II and acetolactate synthase, respectively). Both herbicides are able to control target weeds when applied either pre-emergence or post-emergence [37,38,39]. Compounds **1** and **4** displayed the phytotoxicity in leaf puncture assay at the level comparable with that of metribuzin. Some compounds (**1**, **5**, **6** and **11**) successfully inhibited the growth of lettuce seedlings with ID_50_ less than that of metribuzin and at the same level as metsulfuron-methyl. Among the most phytotoxic compounds **1** and **11** displayed side toxicity, so their further development as new herbicides is not advisable. Mono- (**5**) and bis- (**6**) acetyl derivatives of herbarumin I displayed higher phytotoxicity to lettuce seedlings than their natural precursor (**4**), but remained to be not toxic to microalgae, bacteria and Sf9 cells. Considering the low side toxicity of **5** and **6** it is reasonable to test these compounds for the pre-emergence herbicidal activity soil surface applied or incorporated into the soil.

In pharmaceutical and agrochemical studies, bioavailability or membrane permeability have often been connected to simple molecular descriptors such as logP, molecular weight, or the counts of hydrogen bond acceptors and donors in a molecule. Avram et al. (2014) showed that commercial herbicides obey the following rules: molecular weight 150–500, octanol/ water partition coefficient (hydrophobicity) ≤ 3.5, number of hydrogen bond donors ≤ 3 and number of hydrogen bond acceptors 2–12 [40]. Compounds **1**–**11** meet these requirements and thus demonstrate high pesticide-likeness. The acetylation of natural 10-membered lactones **1**–**4** reasonably increased the lipophilicity of derivatives. The acetylated derivatives **5** and **6** displayed decreased phytotoxicity to sowthistle leaf discs but more potent activity to lettuce seedlings compared to their natural precursor **4**. In leaf-puncture assay the tested compounds are applied to the leaf surface in an aqueous solution, and decrease in water solubility of tested compounds can lead to decrease in its phytotoxic activity. It is confirmed by the negative correlation of logP and leaf-puncture assay data. On the contrary, in seedlings assay the tested compound can be absorbed by roots through direct contact with agar. In that case the increased lipophilicity can lead to the more rapid and effective uptake of compounds into plant tissues. Therefore, the increased phytotoxicity of **5** and **6** to lettuce seedlings compared to herbarumin I (**4**) may be due to the increased uptake across lipophilic plant barriers. However, the increased lipophilicity did not promote the seedlings toxicity of acetyl-derivatives of stagonolide J (compounds **7** and **8**), so, the differences between the phytotoxic activity of 7*S* (**4**, **5**, **6**) and 7*R* (**2**, **7**, **8**) nonenolides are associated with structural features of these compounds and are not correlated with logP.

The qualitative analysis of relationships of structure and phytotoxic activity of some 10-membered lactones was carried out using the leaf puncture assay data [12,16,20]. Evidente et al. (2008) concluded that the functionalization (non-substituted C-2−C-4 moiety and n-propyl residue at C-9) and the conformational freedom of the lactone ring are important for the phytotoxic activity of putaminoxins, pinolidoxins and stagonolides [16]. Similar findings were discovered by Rivero-Cruz et al. (2003), who demonstrated that hydroxylation of the lactone core at C-2 in structurally related herbarumins decreases the phytotoxic effect against seedlings of *A. hypochondriacus* [41]. In our research all the tested natural and semisynthetic ten-membered lactones (**1**–**11**) possess the structural features noted by Evidente et al. (2008) [16] and Rivero-Cruz et al. (2003) [41], but despite this, the described compounds differed in their activity profiles. According to PCA results C-7-oxidized compounds **1**, **10** and **11** formed a separate group. Unlike the other tested nonenolides, they displayed acute toxicity to microalgae, Sf9 cells and bacteria. The different activity profiles of two groups of studied 10-membered lactones indicate the differences in their mode of action (MoA) [42]. The previous SAR studies of ten-membered lactones did not consider the possibility of various mechanisms of phytotoxic action of these natural products, whereas the uniformity of the MoA of compounds included in the library is important for adequate SAR analysis and correct conclusions.

**Figure 4:**
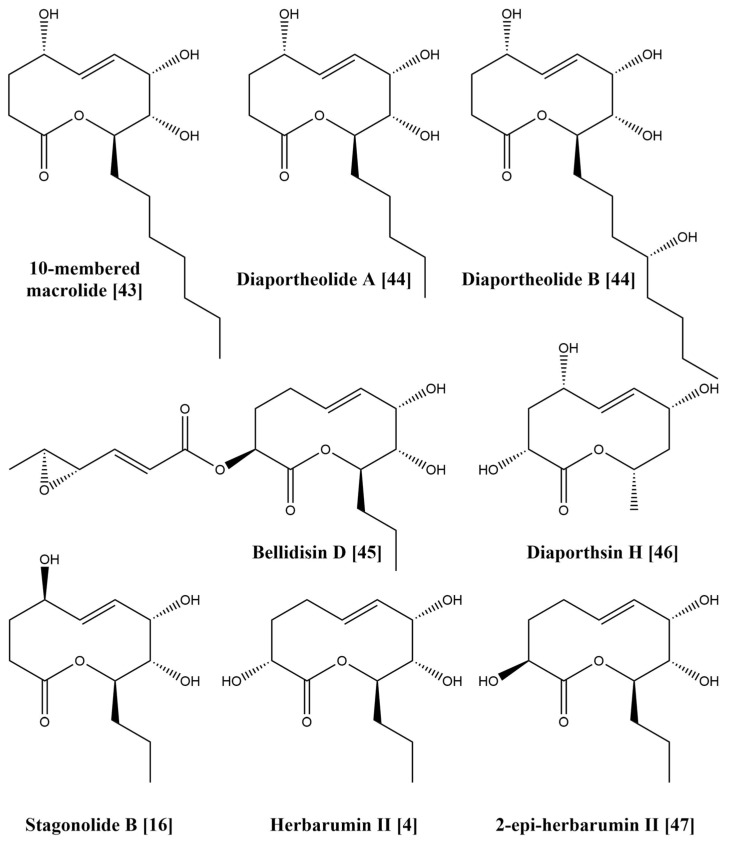
Structurally related fungal ten-membered lactones.

Among the studied 10-membered lactones the plant-specific toxins **2**–**6** and **9** are more potent for natural product-derived herbicides. The characteristic structural features of these compounds are C-7 hydroxyl group and 7*S* configuration. There are related natural products that possess this fragment in their structures, for example recently isolated ten-membered lactone from sponge-associated fungus *Xylaria feejeenisis* [43], diaportheolides A and B from *Diaporthe* sp. SXZ-19 [44] and others [45,46] (Figure 4). Unfortunately, none of these compounds were tested for phytotoxic activity. Such scarce and fragmented information about the biological activity of ten-membered lactones complicates the SAR analysis. The different length of alkyl chain at C-9 and additional hydroxyl groups at the C-2–C-4 site in the structure of these natural products can affect their activity and bioavailability. Stagonolide B [16], herbarumin II [4] and 2-epi-herbarumin II [47] (Figure 4) are the examples of C-2–C-4-modified ten-membered lactones that were tested for phytotoxicity and demonstrated weak or null activity. It is still unclear whether this region should be conserved and non-modified or if the configuration of the substituents at this site also plays a role in the manifestation of phytotoxic activity. This research of phytotoxic ten-membered lactones is intended to intensify the investigation of biological activity of poorly or narrowly studied natural members of this intriguing class of natural products. In further studies we plan to assess the contribution of the C-2–C-4 site and C-5–C-6 double bond in resulting phytotoxic activity using semisynthetic derivatives.

## 5. Conclusions

Four natural ten-membered lactones produced by the fungus *Stagonospora cirsii* G-51 (**1**–**4**) and their seven new semisynthetic derivatives (**5**–**9**) varying at C-7–C-9 site in lactone core were tested for phytotoxic, cytotoxic and antimicrobial activities. Our results indicate that the oxidation of C-7 hydroxyl group to carbonyl resulted in the increase in non-selective toxicity in all used bioassays regardless of the C-9 propyl chain configuration. Using the set of compounds **1**–**11** and the standardized bioassays for phytotoxicity assessment we showed that in non-oxidized ten-membered lactones the configuration of C-7 is critical for phytotoxic activity as the natural and semisynthetic compounds that possess 7*R* configuration (**2**, **7**, **8**) were much less active than their 7*S* analogues (**4**–**6**, respectively). Due to the high inhibitory activity against seedling growth and the lack of side toxicity, acetyl derivatives of herbarumin I (compounds **5** and **6**) are potent for the development of pre-emergent herbicides. Stagonolide A (**1)** and C-7 oxidized stagonolide K (**11)** displayed high phytotoxicity coupled with non-target activity (cytotoxic, antimicrobial), so their further development as new herbicides is not advisable. Different toxicity profiles of C-7 oxidized nonenolides (**1**, **10** and **11**) and compounds **2**–**9** in the performed bioassays suggest the differences in their MoAs despite the structural similarity. The identified structural features of the most active compounds can be used for further rational design of semisynthetic phytotoxic ten-membered lactones as potential natural product-derived herbicides. The results of this study can encourage the future research of the mechanism of phytotoxicity of nonenolides as well as extensive quantitative SAR analysis on an extended set of natural and semisynthetic derivatives.

## Figures and Tables

**Table 1:** Calibration standards for determination of logP.

Compound	t_R_, min	LogP [32]
Imidacloprid (Sigma-Aldrich, St. Louis, MO, USA)	2.59	0.57
Acetamiprid (Sigma-Aldrich)	2.77	0.80
Metribuzin (Sigma-Aldrich)	5.36	1.70
Metsulfuron-methyl (Dr. Ehrenstorfer, Augsburg, Germany)	7.42	2.20
Propiconazole (Sigma-Aldrich)	42.59	3.72
Pyraclostrobin (Sigma-Aldrich)	104.11	3.99

**Table 2:** Biological activity of natural (**1–4**) and semisynthetic (**5**–**11**) nonenolides.

Compound	Phytotoxic Activity				Antimicrobial Activity, *Bacillus subtilis*, Radius of Growth Inhibition Zone, mm	Cytotoxic Activity, Sf9 Cells, LC_50_, μg/mL (μM)	LogP (HPLC-RP Method)
	Sowthistle Leaves, Necrosis Diameter, mm		Lettuce Seedlings, ID_50_, 10^−8^ mole/seed	*Haematococcus* *lacustris*, LC_50_, μg/mL (μM)			
	48 h	120 h					
**1**	6.3 ± 0.4 ^f *^	6.4 ± 0.3 ^g^	3	1 (4.4)	16.0 ± 1.0	3 (13)	1.87
**2**	1.6 ± 0.5 ^d^	4.2 ± 0.4 ^bcd^	n/a	n/a	0	n/a	1.31
**3**	4.3 ± 0.2 ^c^	4.9 ± 0.3 ^de^	94	n/a	0	n/a	1.73
**4**	2.8 ± 0.2 ^b^	4.8 ± 0.3 ^cde^	35	n/a	0	n/a	1.39
**5**	3.7 ± 0.3 ^c^	4.0 ± 0.3 ^bc^	4	n/a	0	n/a	2.32
**6**	0.3 ± 0.2 ^a^	0.5 ± 0.2 ^a^	7	n/a	0	n/a	3.18
**7**	0 ^a^	0 ^a^	n/a	n/a	0	n/a	2.22
**8**	0 ^a^	0 ^a^	n/a	n/a	0	n/a	3.20
**9**	2.6 ± 0.4 ^b^	2.6 ± 0.4 ^f^	n/a	n/a	0	88 (346.4)	3.31
**10**	3.8 ± 0.3 ^c^	3.9 ± 0.3 ^b^	30	3 (11.2)	8.3 ± 0.6	6 (22.4)	2.51
**11**	5.3 ± 0.3 ^e^	5.3 ± 0.3 ^e^	4	2 (9.5)	14.0 ± 1.0	5 (23.7)	2.23
**metribuzin**	4.4 ± 0.5 ^c^	5.4 ± 0.7 ^e^	18	2 (9.3)	n/t	n/t	1.70 [32]
**metsulfuron-methyl**	0 ^a^	0 ^a^	3	>50 (>131.0)	n/t	n/t	2.20 [32]

* Letters indicate statistically significant differences (*p*=0.05), n/a—not active, n/t—not tested.

## Supplementary Materials

The following are available online at https://www.mdpi.com/article/10.3390/jof7100829/s1, Figure S1: ^1^H NMR spectrum of compound **5**, Figure S2: ^13^C NMR spectrum of compound **5**, Figure S3: COSY spectrum of compound **5**, **Figure S4**: HMBC spectrum of compound **5**, Figure S5: HSQC spectrum of compound **5**, Figure S6: TOCSY spectrum of compound **5**, Figure S7: ESIMS of compound **5** recorded in positive ion mode, Figure S8: ^1^H NMR spectrum of compound **6**, Figure S9: ^13^C NMR spectrum of compound **6**, Figure S10: DEPT spectrum of compound **6**, Figure S11: COSY spectrum of compound **6**, Figure S12: HMBC spectrum of compound **6**, Figure S13: HSQC spectrum of compound **6**, Figure S14: ESIMS of compound **6** recorded in positive ion mode, Figure S15: ^1^H NMR spectrum of compound **7**, Figure S16: ^13^C NMR spectrum of compound **7**, Figure S17: COSY spectrum of compound **7**, Figure S18: NOESY spectrum of compound **7**, Figure S19: HMBC spectrum of compound **7**, Figure S20: HSQC spectrum of compound **7**, Figure S21: ESIMS of compound **7** recorded in positive ion mode, Figure S22: ^1^H NMR spectrum of compound **8**, Figure S23: ^13^C NMR spectrum of compound **8**, Figure S24: COSY spectrum of compound **8**, Figure S25: NOESY spectrum of compound **8**, Figure S26: HMBC spectrum of compound **8**, Figure S27: HSQC spectrum of compound **8**, Figure S28: ESIMS of compound **8** recorded in positive ion mode, Figure S29: ^1^H NMR spectrum of compound **9**, Figure S30: ^13^C NMR spectrum of compound **9**, Figure S31: COSY spectrum of compound **9**, Figure S32: HMBC spectrum of compound **9**, Figure S33: HSQC spectrum of compound **9**, Figure S34: TOCSY spectrum of compound **9**, Figure S35: ESIMS of compound **9** recorded in positive ion mode, Figure S36: ^1^H NMR spectrum of compound **10**, Figure S37: ^13^C NMR spectrum of compound **10**, Figure S38: COSY spectrum of compound **10**, Figure S39: HMBC spectrum of compound **10**, Figure S40: HSQC spectrum of compound **10**, Figure S41: ESIMS of compound **10** recorded in positive ion mode, Figure S42: ^1^H NMR spectrum of compound **11**, Figure S43: ^13^C NMR spectrum of compound **11**, Figure S44: COSY spectrum of compound **11**, Figure S45: HMBC spectrum of compound **11**, Figure S46: HSQC spectrum of compound **11**, Figure S47: ESIMS of compound **11** recorded in positive ion mode, Figure S48: Phytotoxic activity of compounds **1**–**11** on sowthistle punctured leaf discs at 120 h after treatment.
