# Supplementary material for: Structure–Activity Relationship of Phytotoxic Natural 10-Membered Lactones and Their Semisynthetic Derivatives
Source: J Fungi (Basel). 2021 Oct 3;7(10):829. doi: 10.3390/jof7100829 (PMC8541495; doi:10.3390/jof7100829)
Supplement: Supplementary file 1 [file jof-07-00829-s001.zip › jof-1411440-supplementary.pdf]

Structure–Activity Relationship of Phytotoxic Natural 10-Membered Lactones and Their Semisynthetic Derivatives

Anna Dalinova*[1], Anatoly Fedorov[1], Vsevolod Dubovik[1], Olga Voitsekhovskaja[2], Elena Tyutereva[2], Sergey Smirnov[3], Dmitry Kochura[4], Leonid Chisty[4], Igor Senderskiy[1], Alexander Berestetskiy[1]

[1] All-Russian Institute of Plant Protection, Saint Petersburg, Pushkin 196608, Russia; adalinova@vizr.spb.ru (A.D.); afedorov@vizr.spb.ru (A.F.); vdubovik@vizr.spb.ru (V.D.); senderskiy@mail.ru (I.S.);
aberestetskiy@vizr.spb.ru (A.B.)

[2] Komarov Botanical Institute, Russian Academy of Sciences, Saint Petersburg 197376, Russia; ovoitse@binran.ru (O.V.); tuterlena@mail.ru (E.T.)

[3] St. Petersburg State University, Saint Petersburg 198504, Russia; sergey.smirnov@spbu.ru

[4] Research Institute of Hygiene, Occupational Pathology and Human Ecology, Federal Medical Biological Agency, Saint-Petersburg 188663, Russia; 89117050635@yandex.ru (D.K.); mehrn.q2@gmail.com (L.C.)

* Correspondence: adalinova@vizr.spb.ru

Figure S1 – ^{1}H NMR spectrum of compound **5**

Figure S2 – ^{13}C NMR spectrum of compound **5**

Figure S3 – COSY spectrum of compound **5**

Figure S4 – HMBC spectrum of compound **5**

Figure S5 – HSQC spectrum of compound **5**

Figure S6 – TOCSY spectrum of compound **5**

Figure S7 – ESIMS of compound **5** recorded in positive ion mode

Figure S8 – ^{1}H NMR spectrum of compound **6**

Figure S9 – ^{13}C NMR spectrum of compound **6**

Figure S10 – DEPT spectrum of compound **6**

Figure S11 – COSY spectrum of compound **6**

Figure S12 – HMBC spectrum of compound **6**

Figure S13 – HSQC spectrum of compound **6**

Figure S14 – ESIMS of compound **6** recorded in positive ion mode

Figure S15 – ^{1}H NMR spectrum of compound **7**

Figure S16 – ^{13}C NMR spectrum of compound **7**

Figure S17 – COSY spectrum of compound **7**

Figure S18 – NOESY spectrum of compound **7**

Figure S19 – HMBC spectrum of compound **7**

Figure S20 – HSQC spectrum of compound **7**

Figure S21 – ESIMS of compound **7** recorded in positive ion mode

Figure S22 – ^{1}H NMR spectrum of compound **8**

Figure S23 – ^{13}C NMR spectrum of compound **8**

Figure S24 – COSY spectrum of compound **8**

Figure S25 – NOESY spectrum of compound **8**

Figure S26 – HMBC spectrum of compound **8**

Figure S27 – HSQC spectrum of compound **8**

Figure S28 – ESIMS of compound **8** recorded in positive ion mode

Figure S29 – ^{1}H NMR spectrum of compound **9**

Figure S30 – ^{13}C NMR spectrum of compound **9**

Figure S31 – COSY spectrum of compound **9**

Figure S32 – HMBC spectrum of compound **9**

Figure S33 – HSQC spectrum of compound **9**

Figure S34 – TOCSY spectrum of compound **9**

Figure S35 – ESIMS of compound **9** recorded in positive ion mode

Figure S36 – ^{1}H NMR spectrum of compound **10**

Figure S37 –^{13}C NMR spectrum of compound **10**

Figure S38 – COSY spectrum of compound **10**

Figure S39 – HMBC spectrum of compound **10**

Figure S40 – HSQC spectrum of compound **10**

Figure S41 – ESIMS of compound **10** recorded in positive ion mode

Figure S42 – ^{1}H NMR spectrum of compound **11**

Figure S43 –^{13}C NMR spectrum of compound **11**

Figure S44 – COSY spectrum of compound **11**

Figure S45– HMBC spectrum of compound **11**

Figure S46 – HSQC spectrum of compound **11**

Figure S47 – ESIMS of compound **11** recorded in positive ion mode

Figure S48 – Phytotoxic activity of compounds **1** – **11** on sowthistle punctured leaf discs at 120 hours after treatment

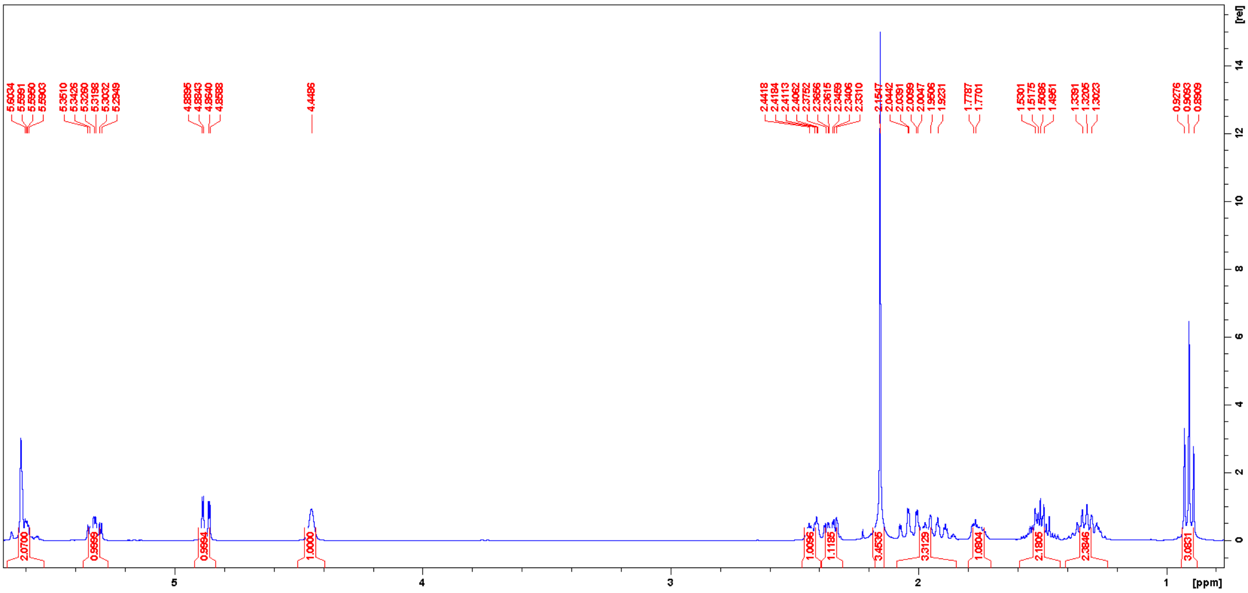

Figure S1 – ^{1}H NMR spectrum of compound **5**

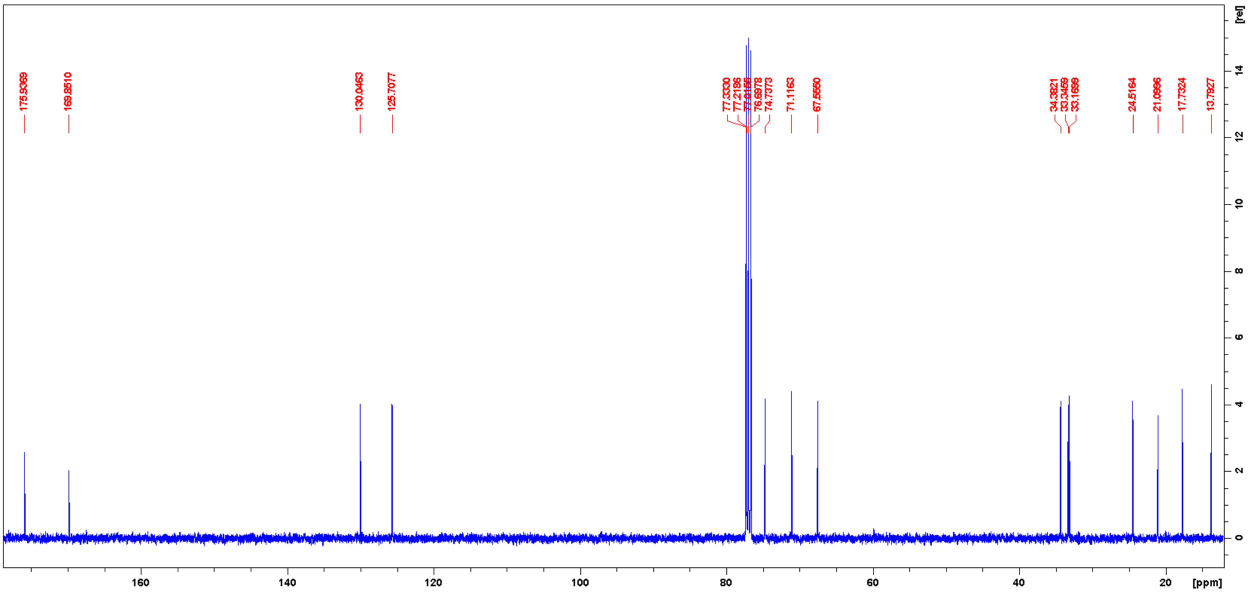

Figure S2 – ^{13}C NMR spectrum of compound **5**

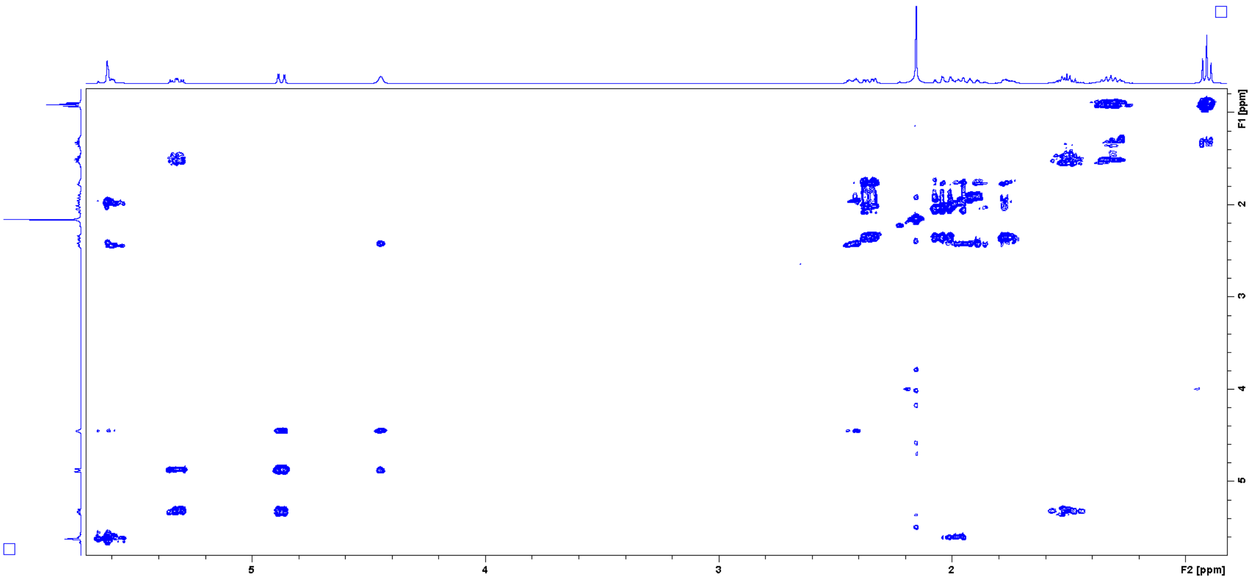

Figure S3 – COSY spectrum of compound **5**

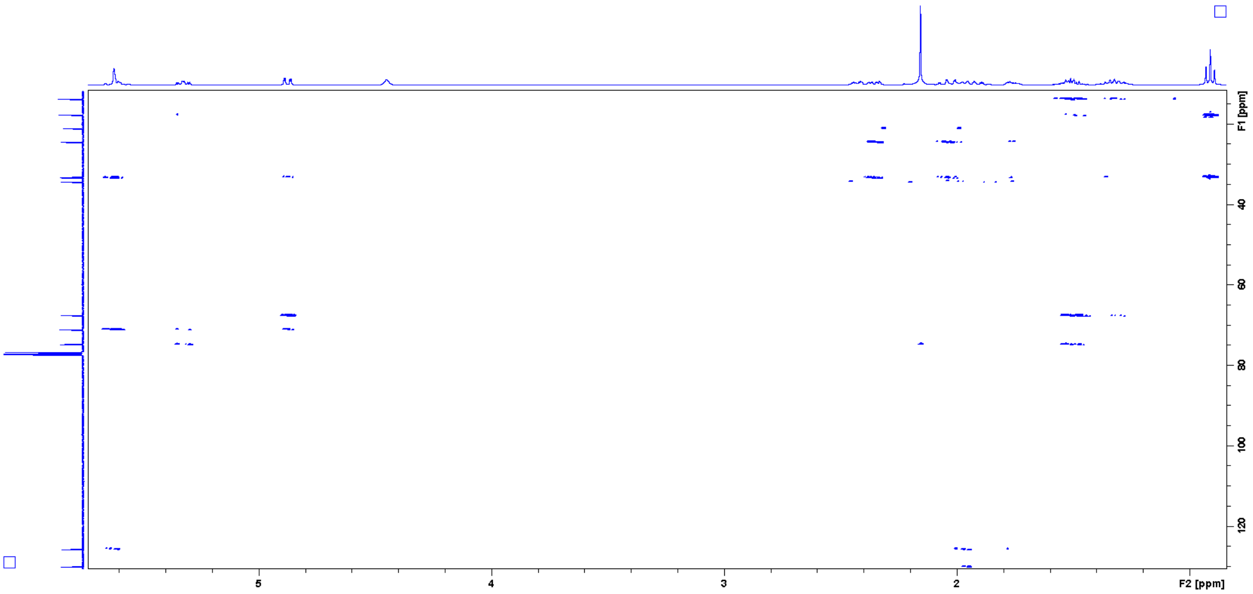

Figure S4 – HMBC spectrum of compound **5**

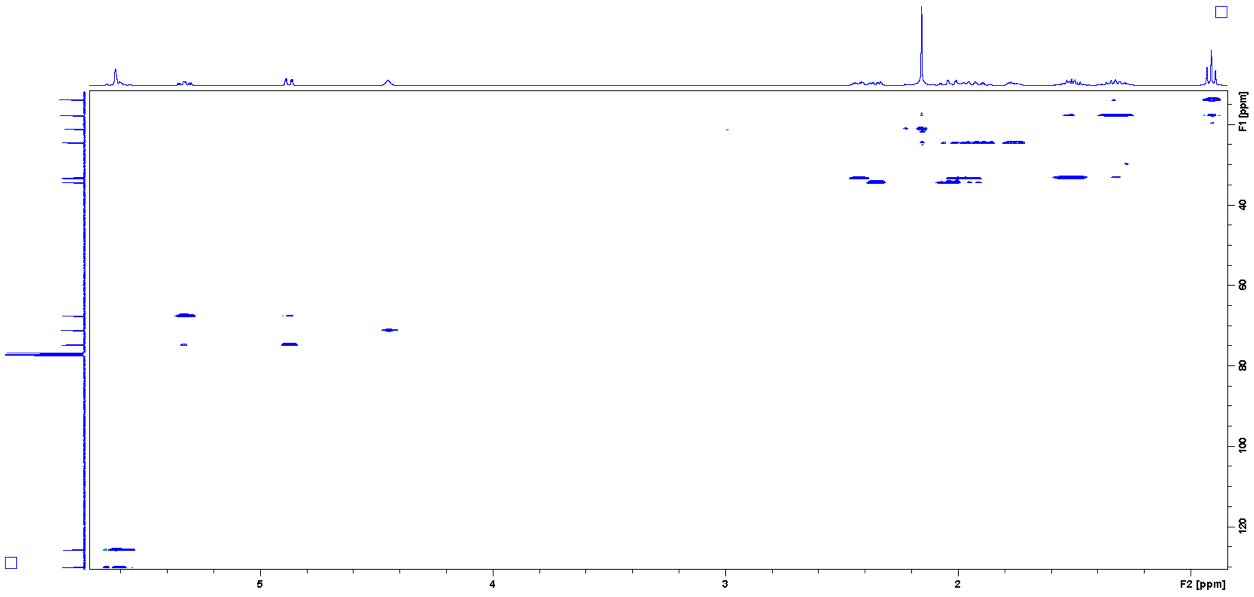

Figure S5 – HSQC spectrum of compound **5**

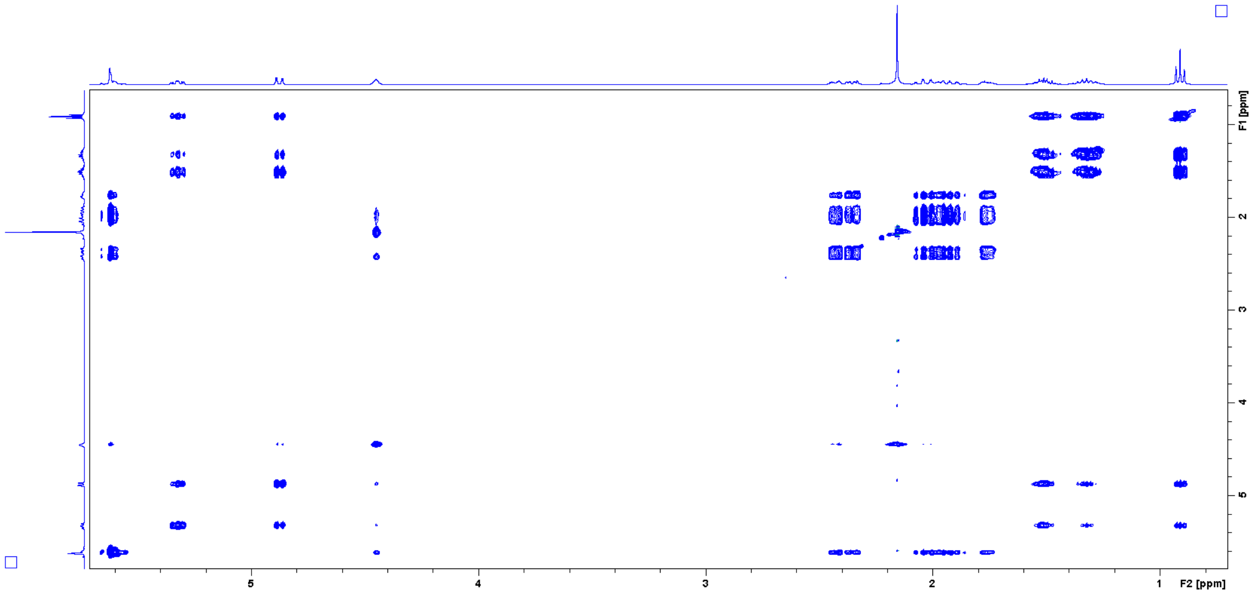

Figure S6 – TOCSY spectrum of compound **5**

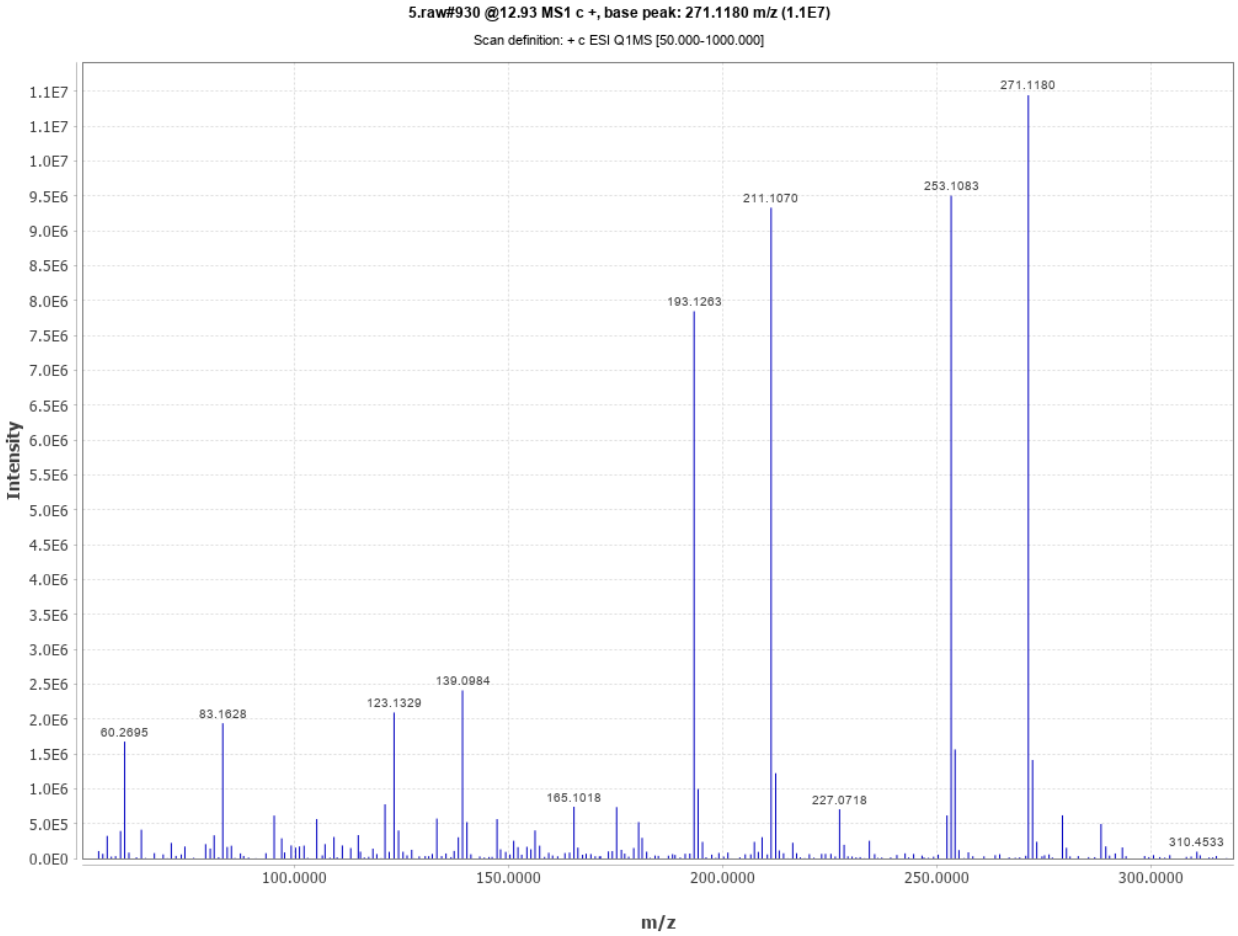

Figure S7 – ESIMS of compound **5** recorded in positive ion mode

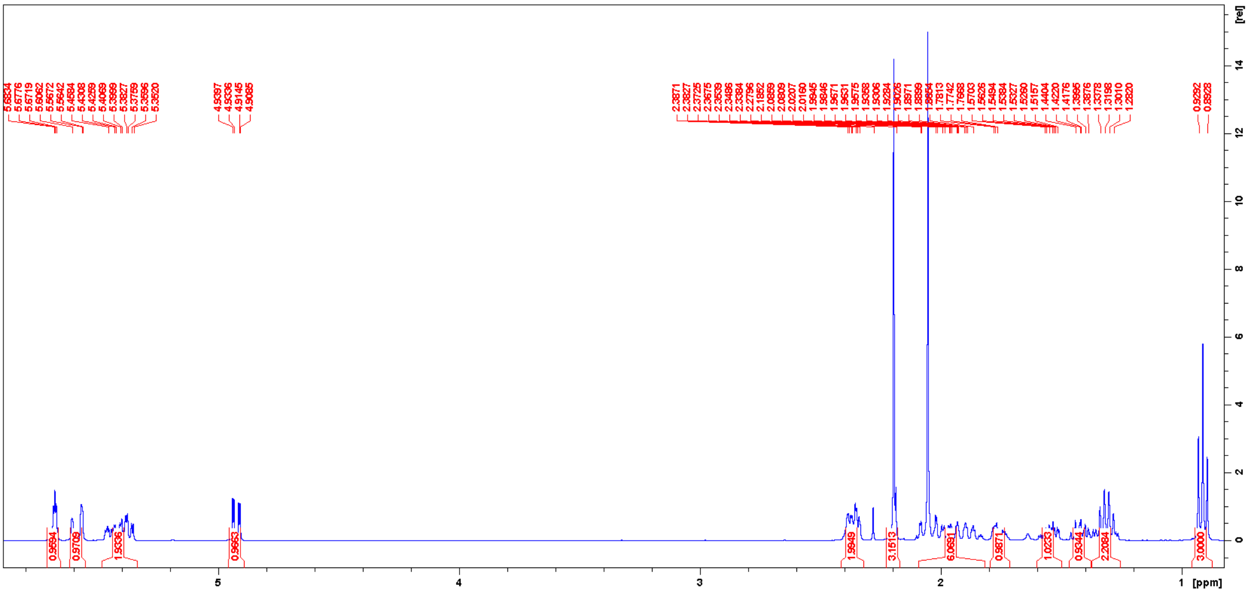

Figure S8 – ^{1}H NMR spectrum of compound **6**

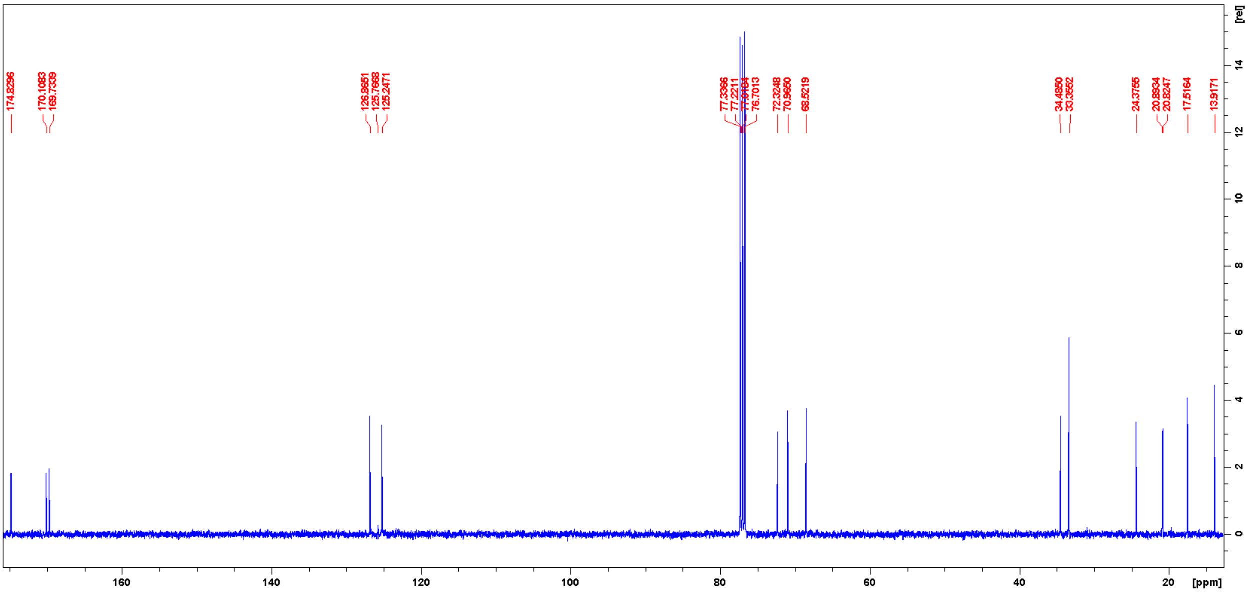

Figure S9 – ^{13}C NMR spectrum of compound **6**

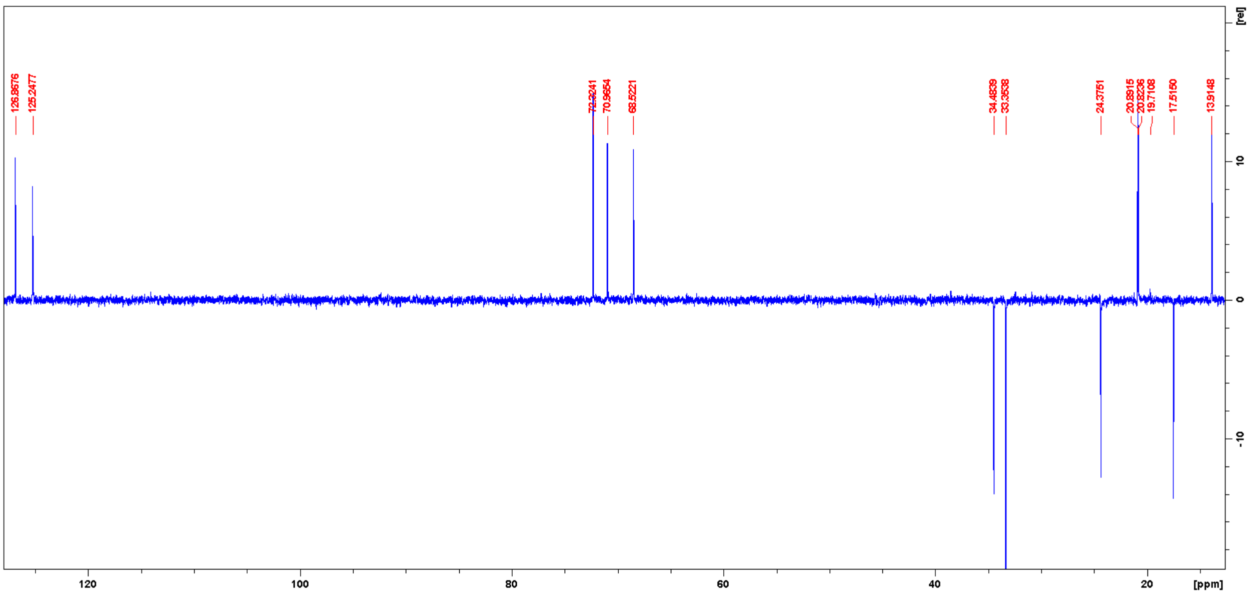

Figure S10 – DEPT spectrum of compound **6**

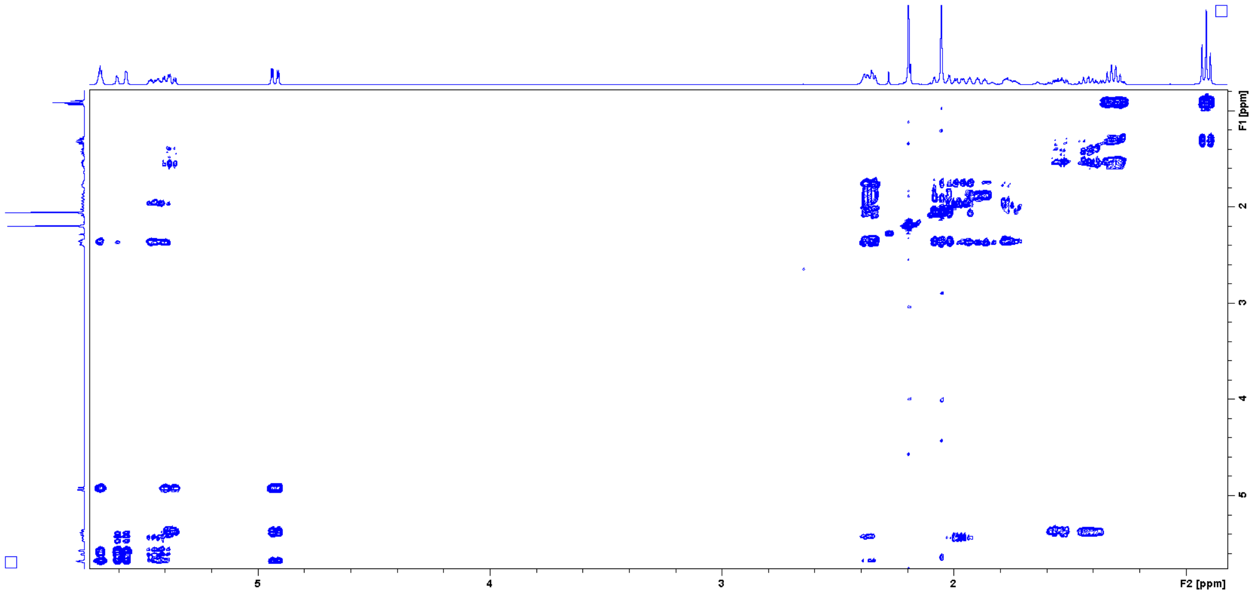

Figure S11 – COSY spectrum of compound **6**

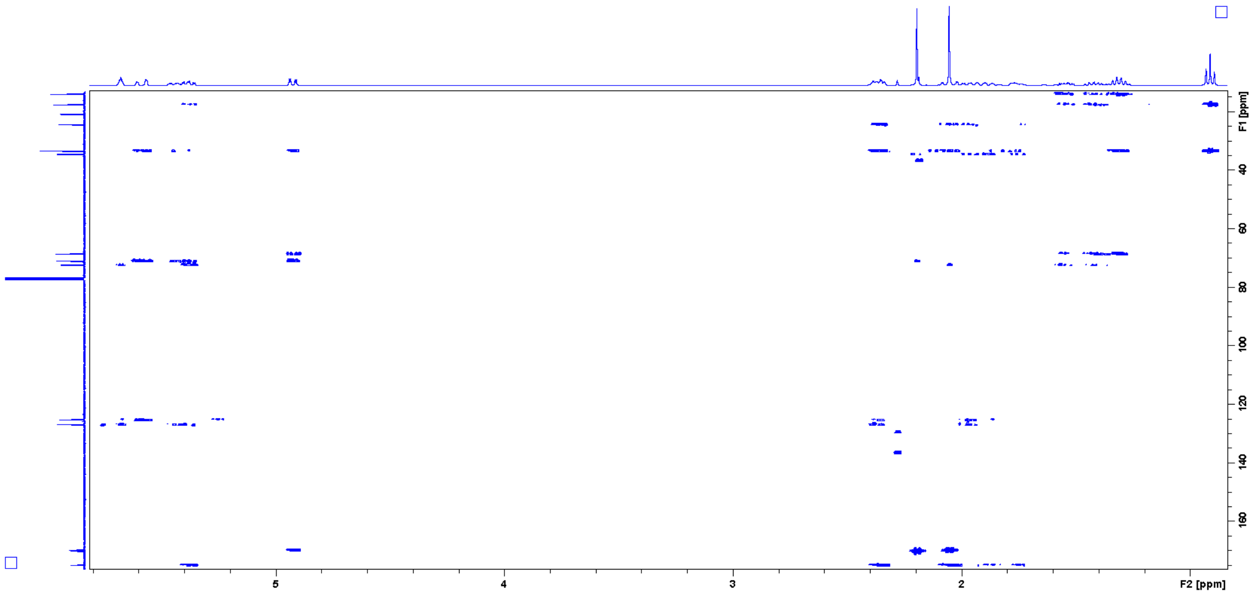

Figure S12 – HMBC spectrum of compound **6**

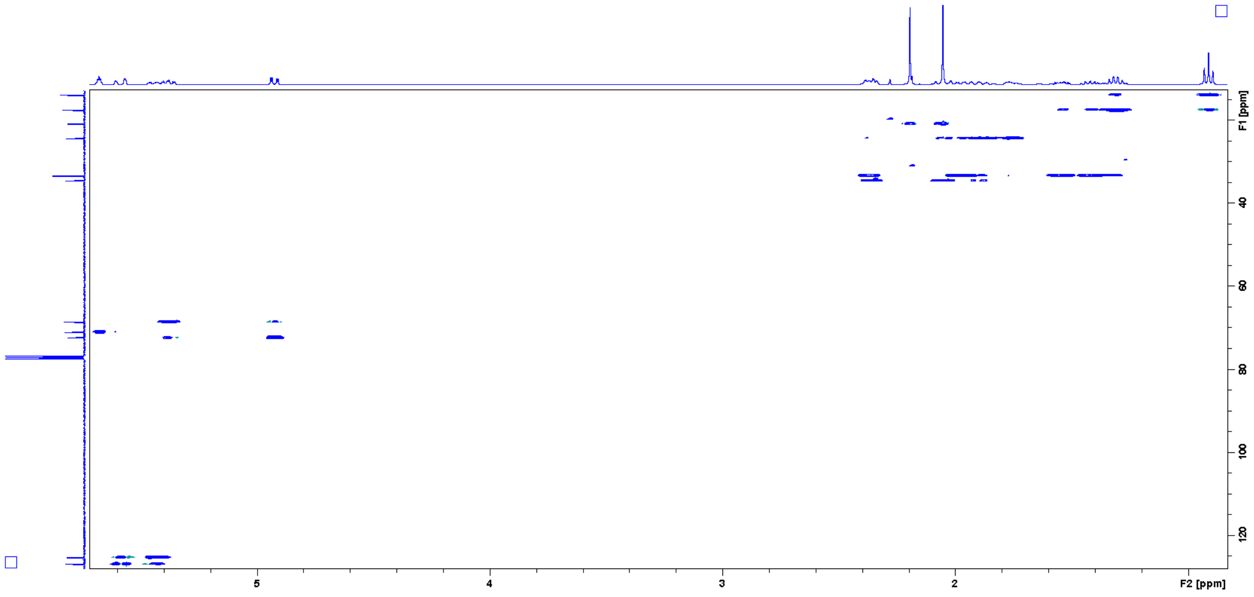

Figure S13 – HSQC spectrum of compound **6**

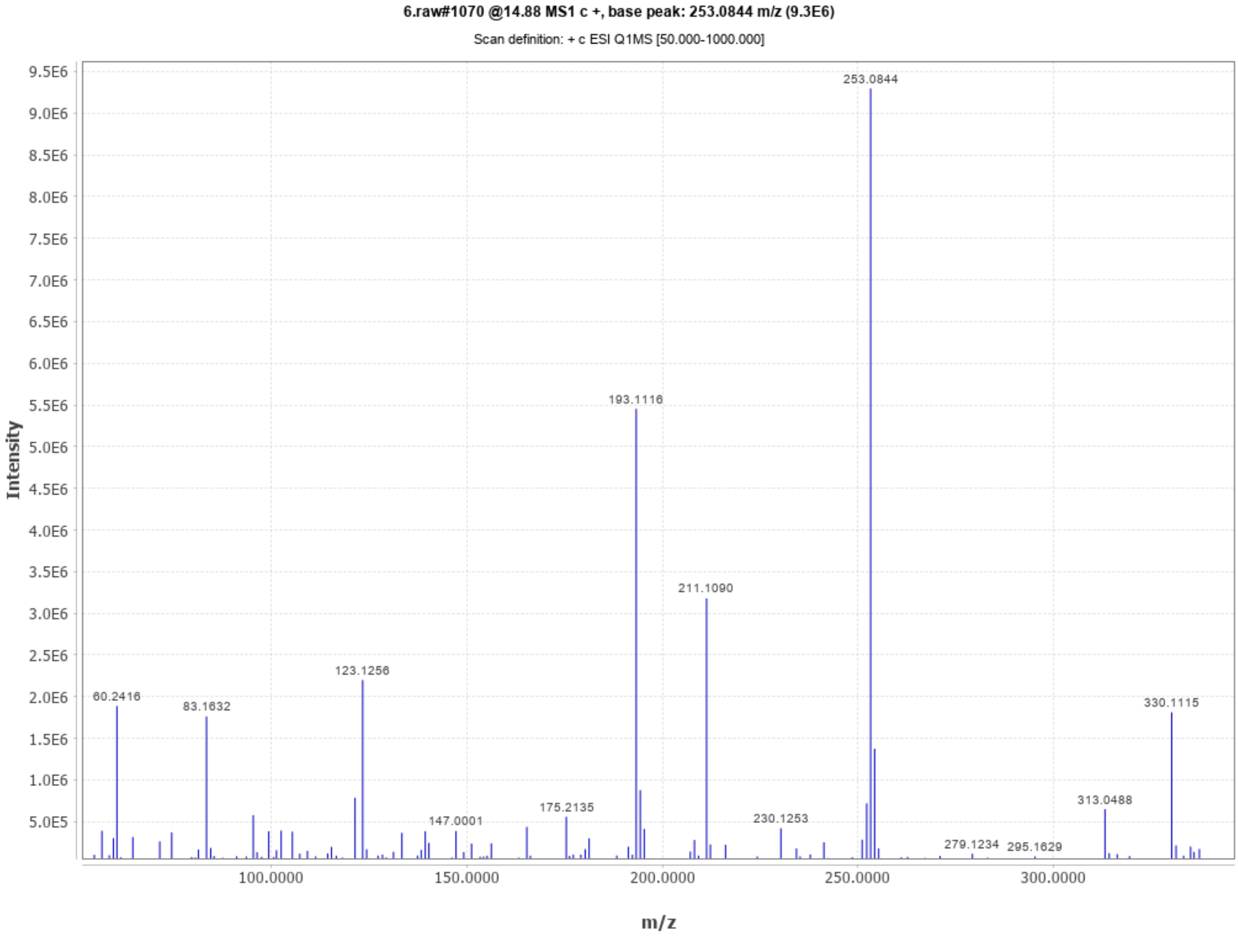

Figure S14 – ESIMS of compound **6** recorded in positive ion mode

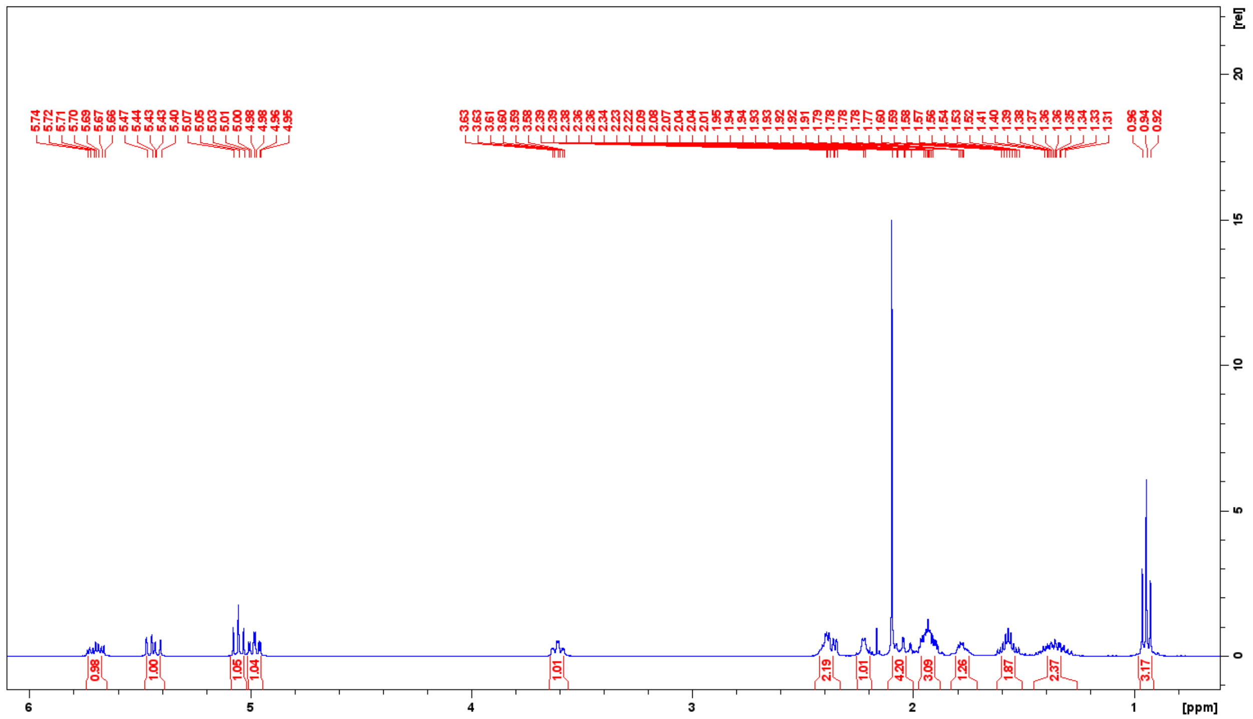

Figure S15 – ^{1}H NMR spectrum of compound **7**

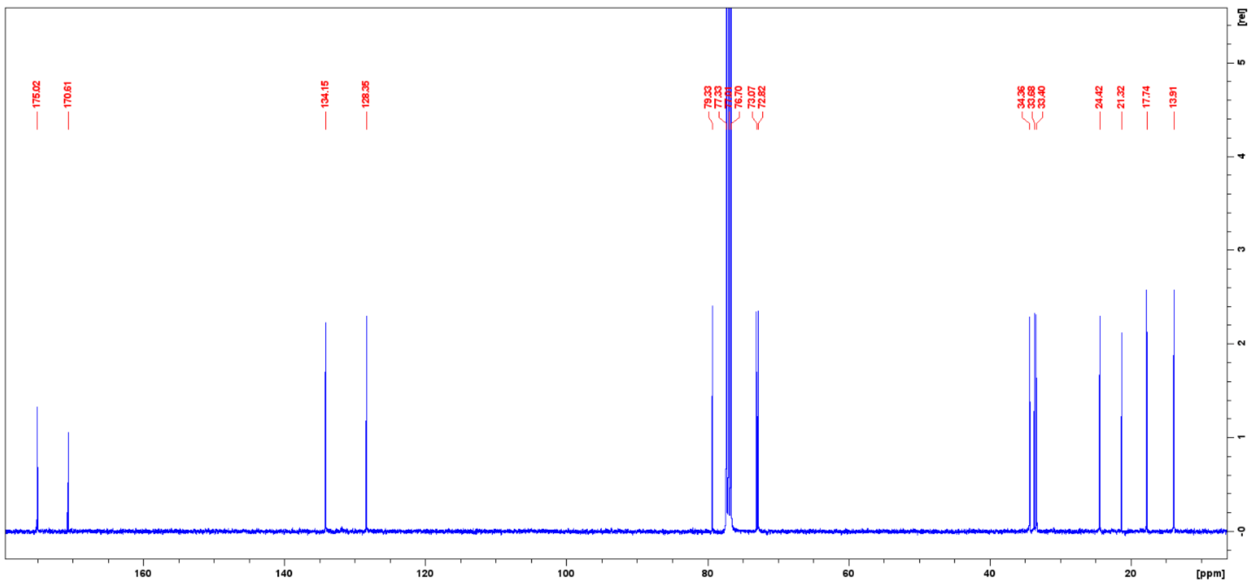

Figure S16 – ^{13}C NMR spectrum of compound **7**

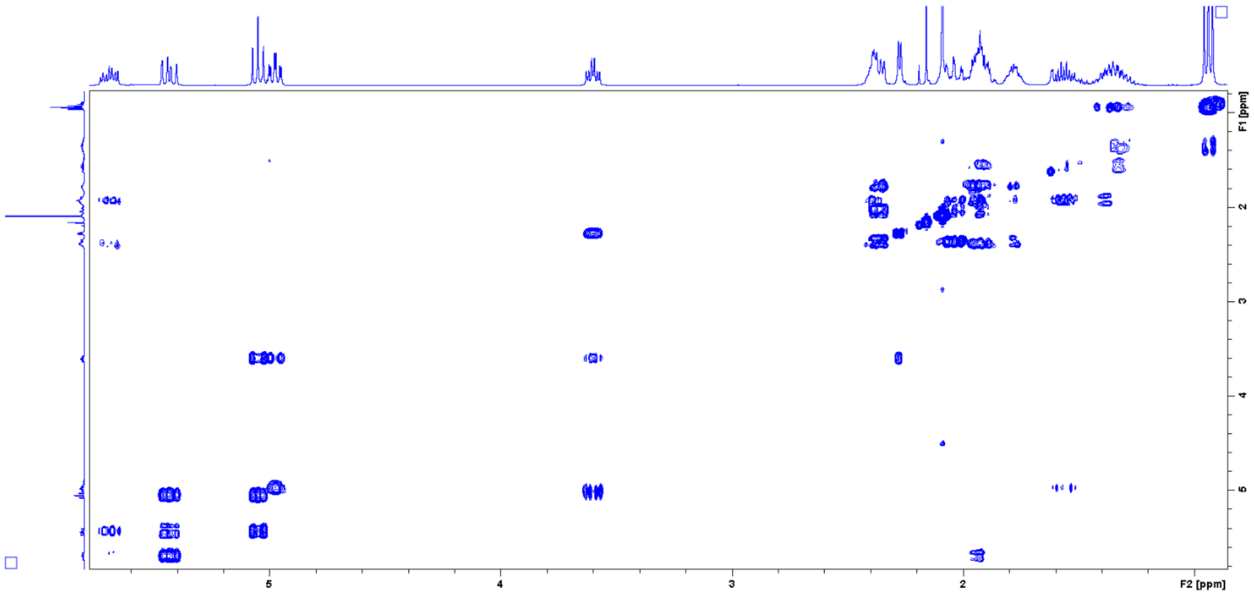

Figure S17 – COSY spectrum of compound **7**

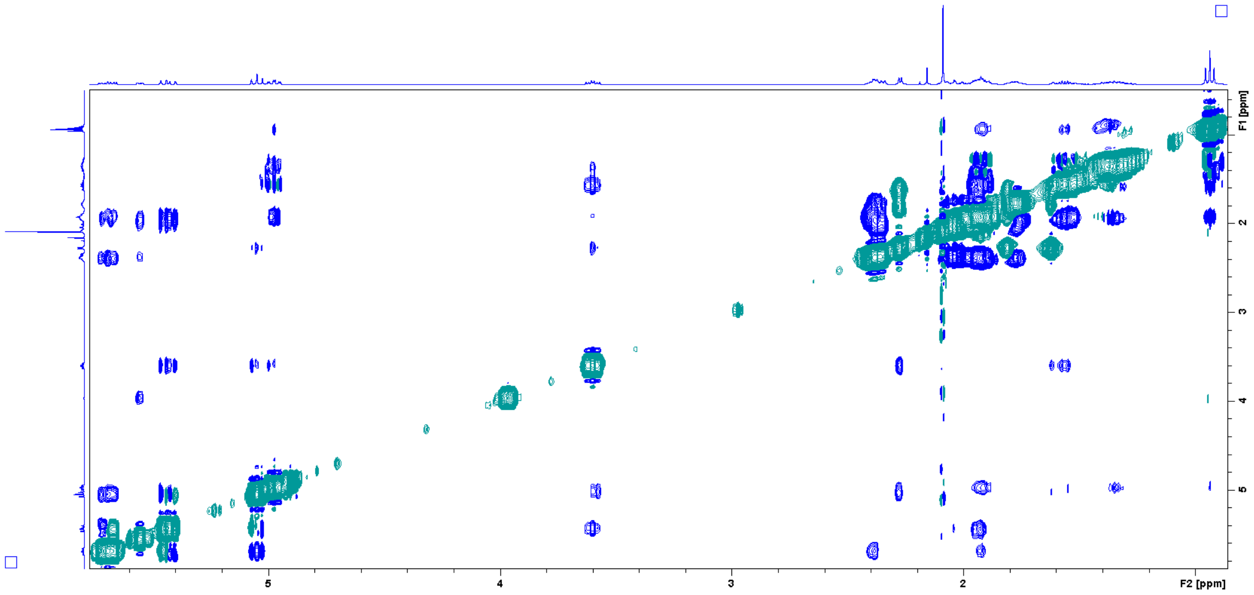

Figure S18 – NOESY spectrum of compound **7**

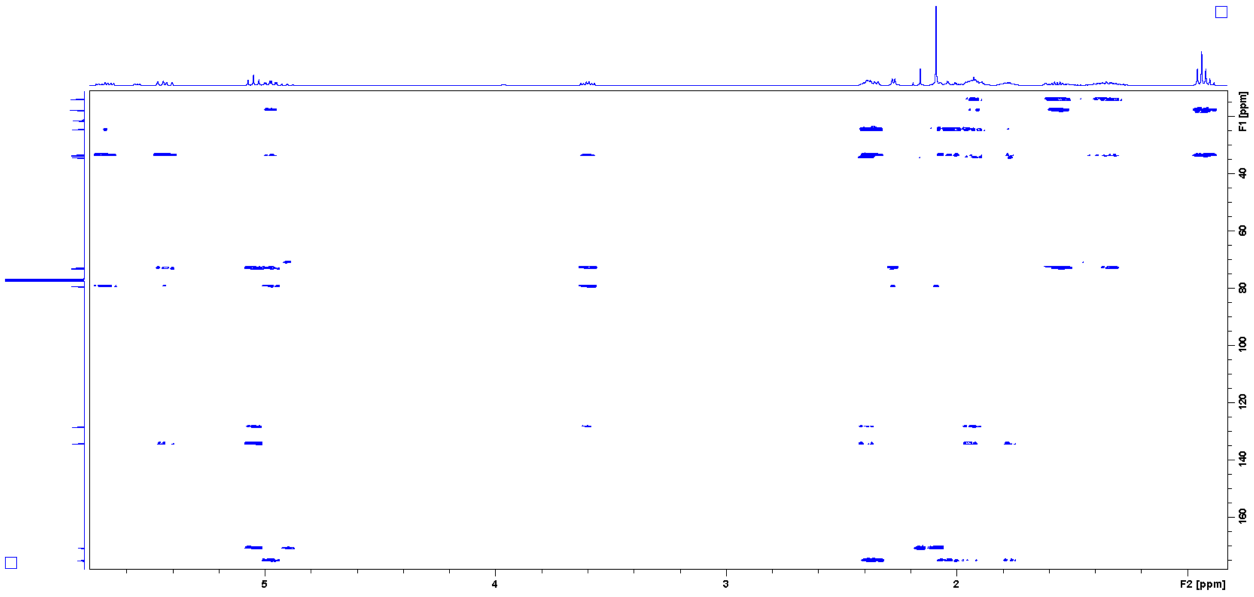

Figure S19 – HMBC spectrum of compound **7**

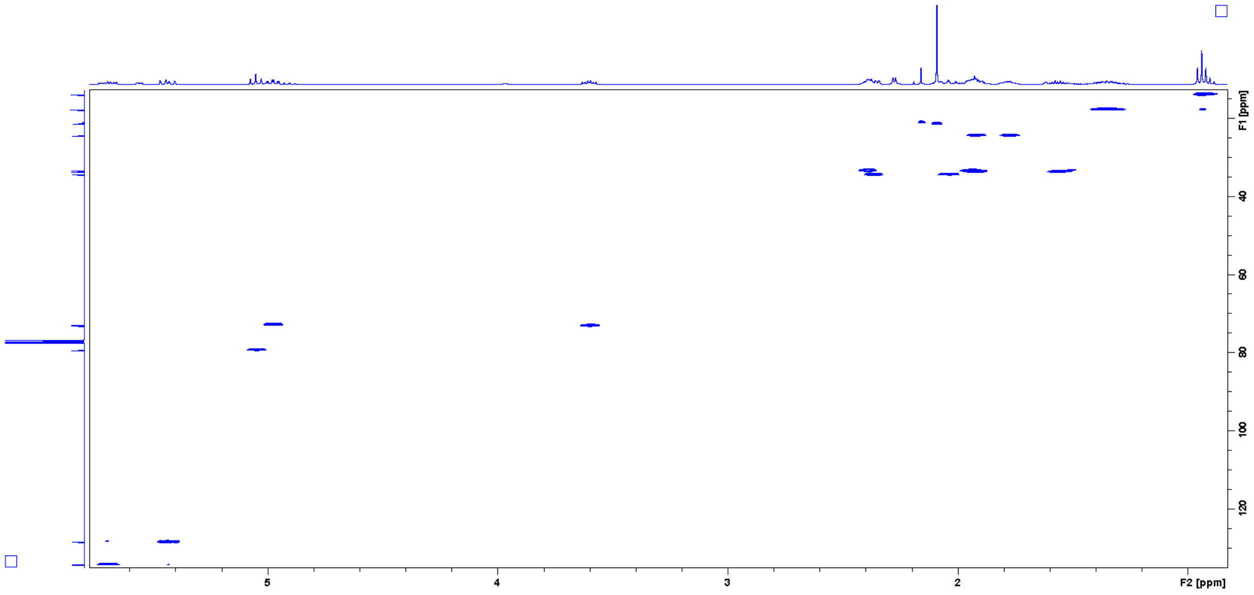

Figure S20 – HSQC spectrum of compound **7**

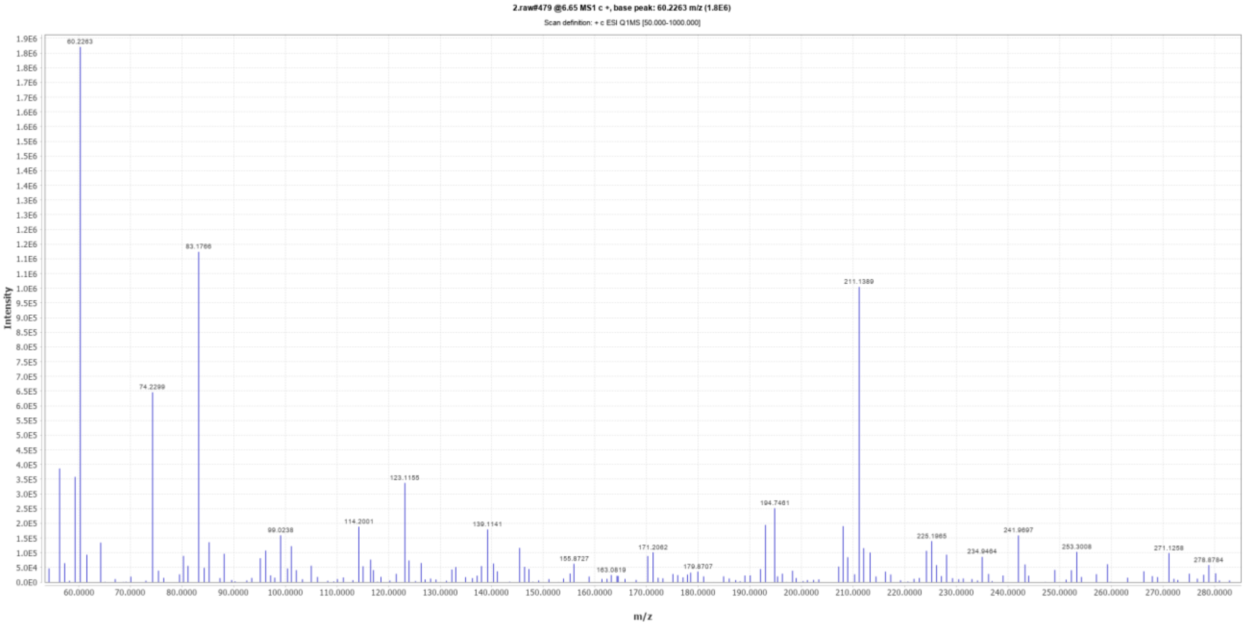

Figure S21 – ESIMS of compound **7** recorded in positive ion mode

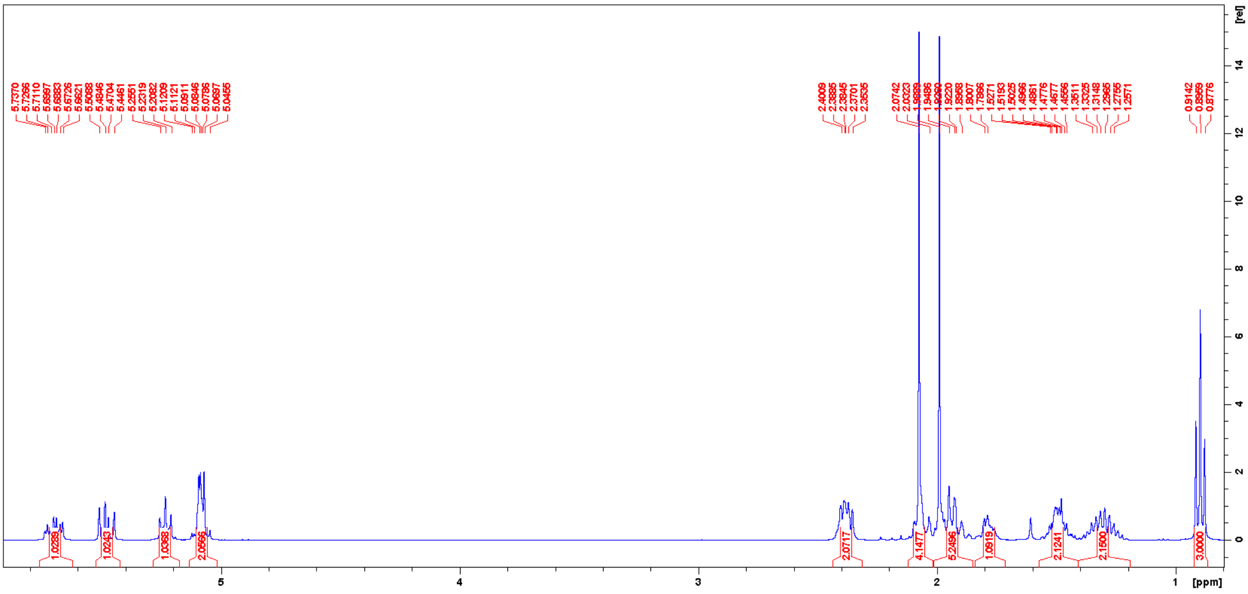

Figure S22 – ^{1}H NMR spectrum of compound **8**

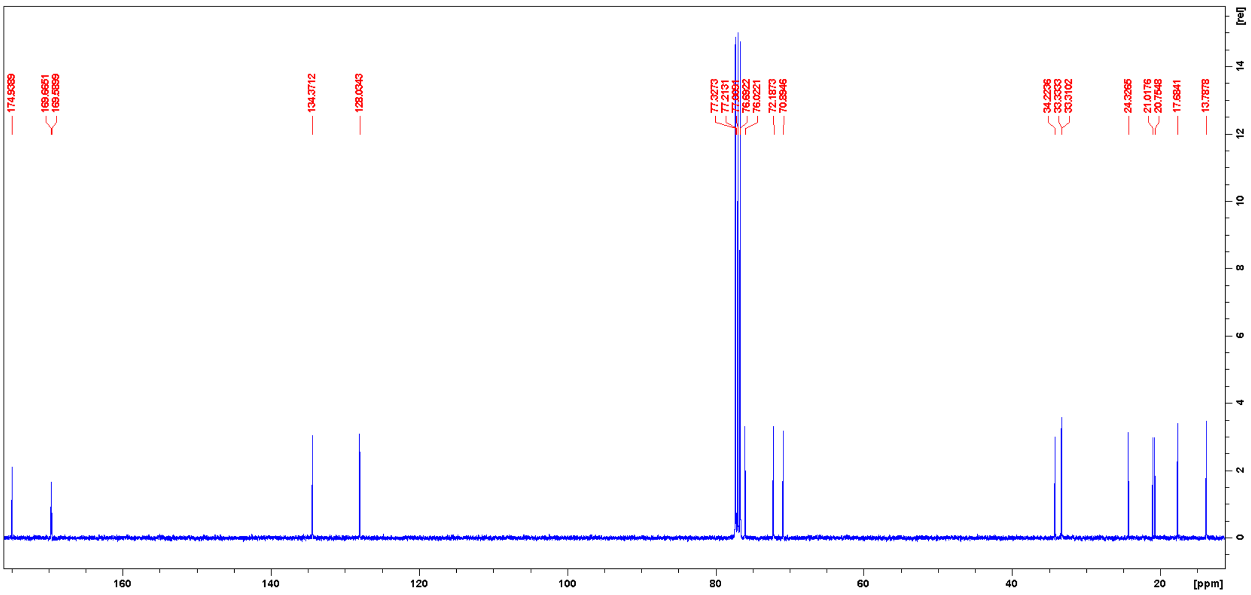

Figure S23 – ^{13}C NMR spectrum of compound **8**

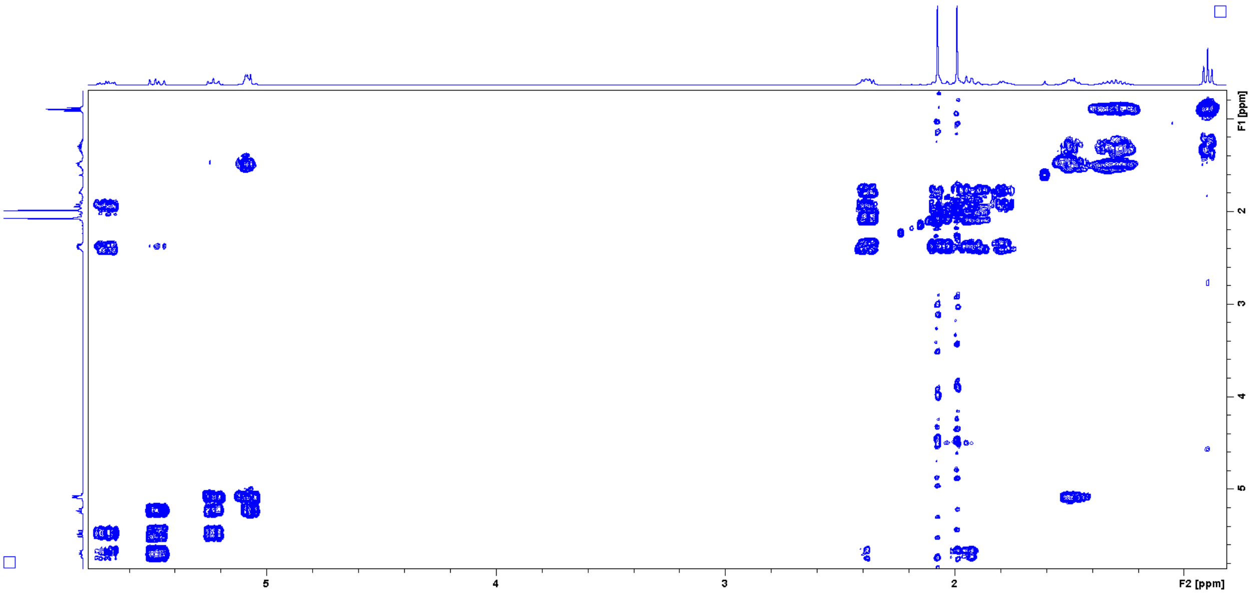

Figure S24 – COSY spectrum of compound **8**

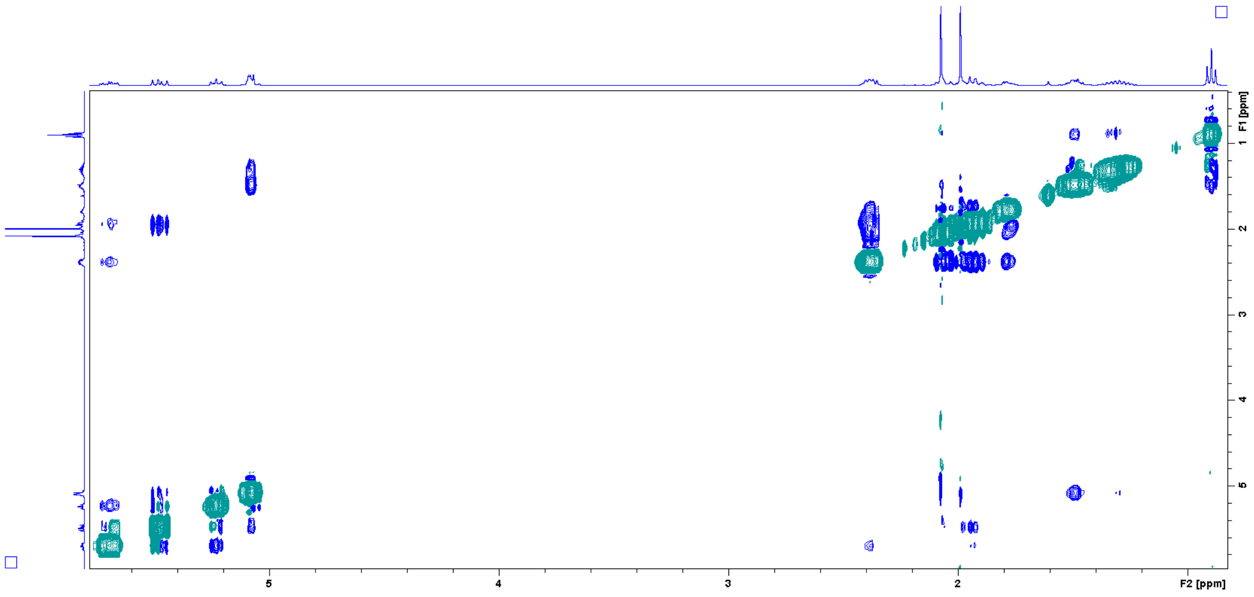

Figure S25 – NOESY spectrum of compound **8**

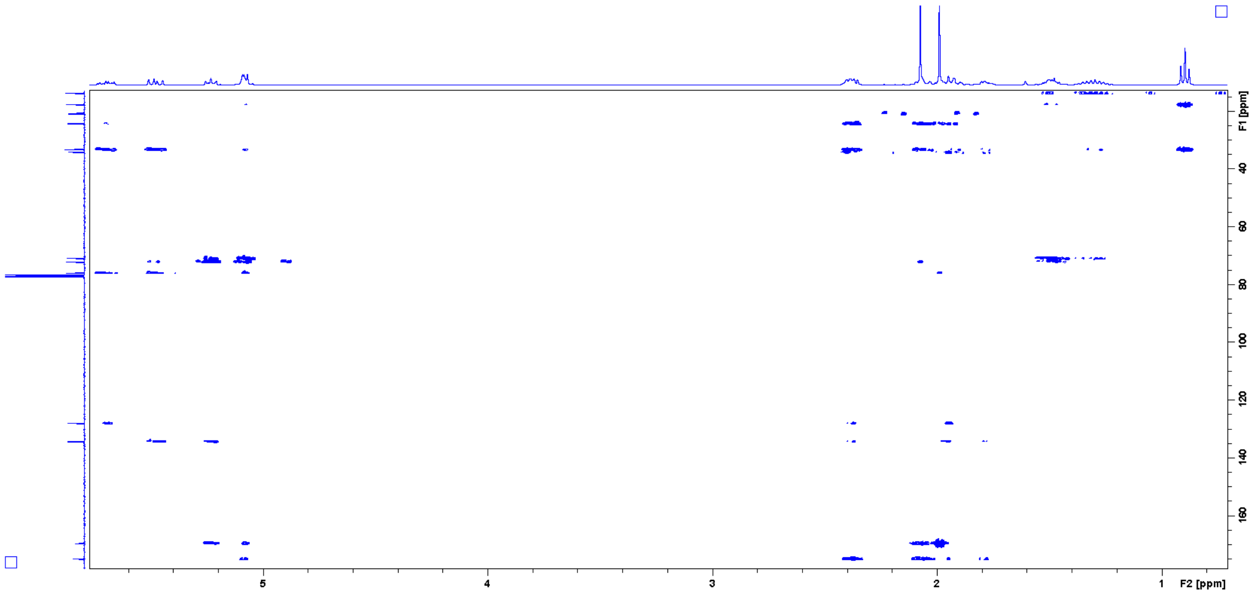

Figure S26 – HMBC spectrum of compound **8**

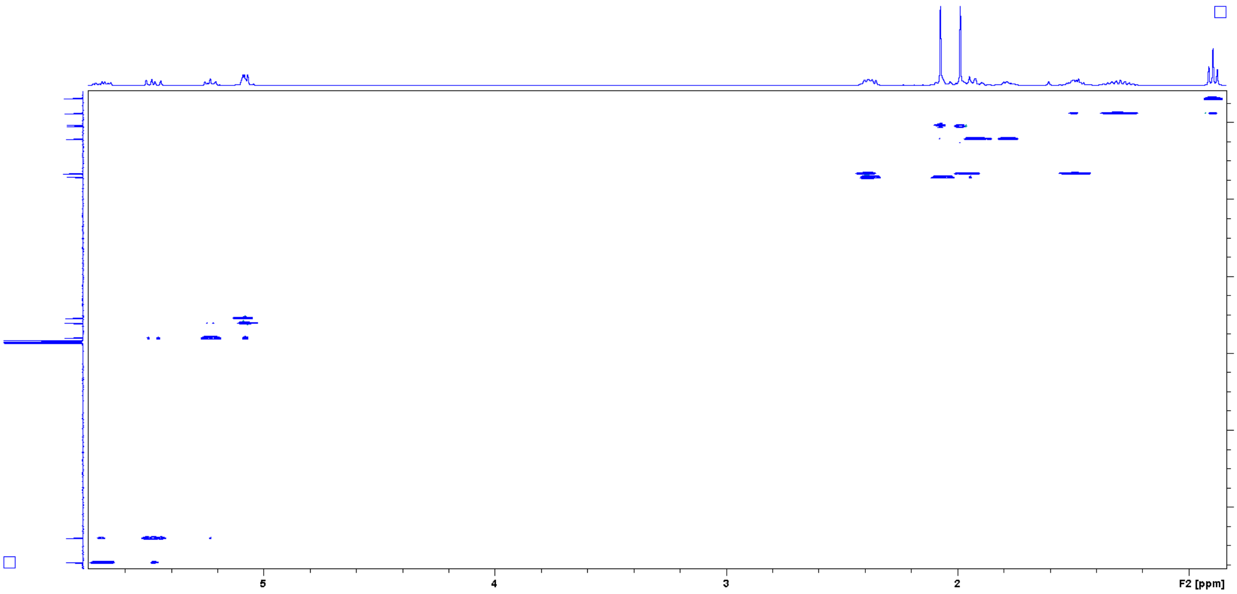

Figure S27 – HSQC spectrum of compound **8**

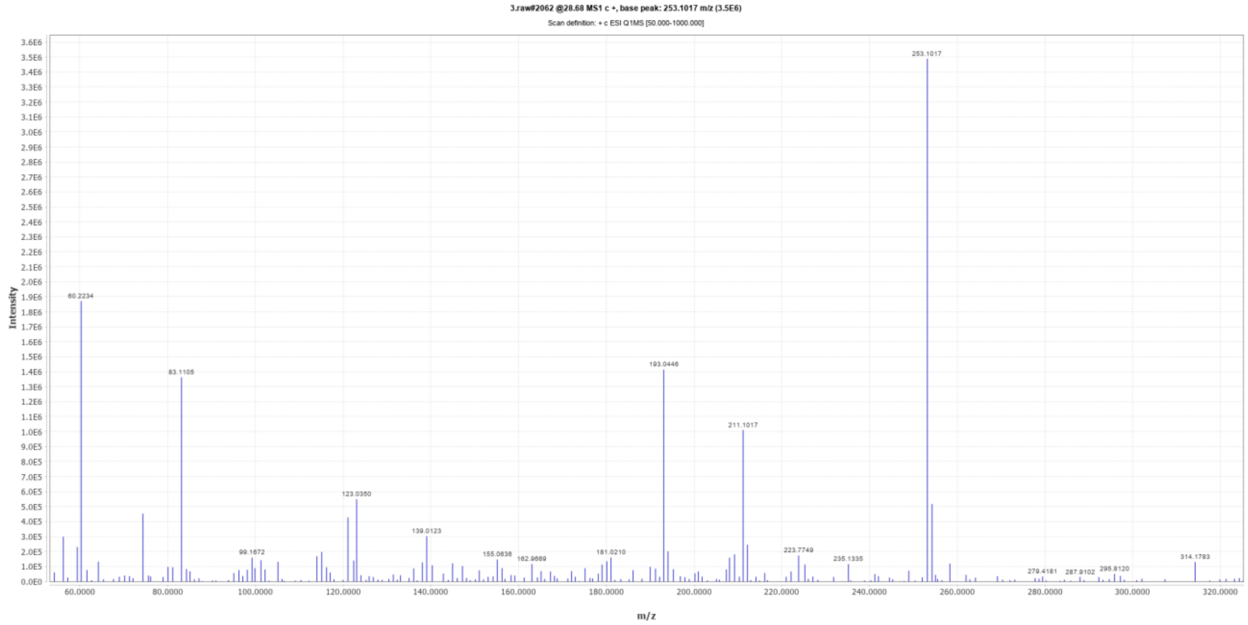

Figure S28 – ESIMS of compound **8** recorded in positive ion mode

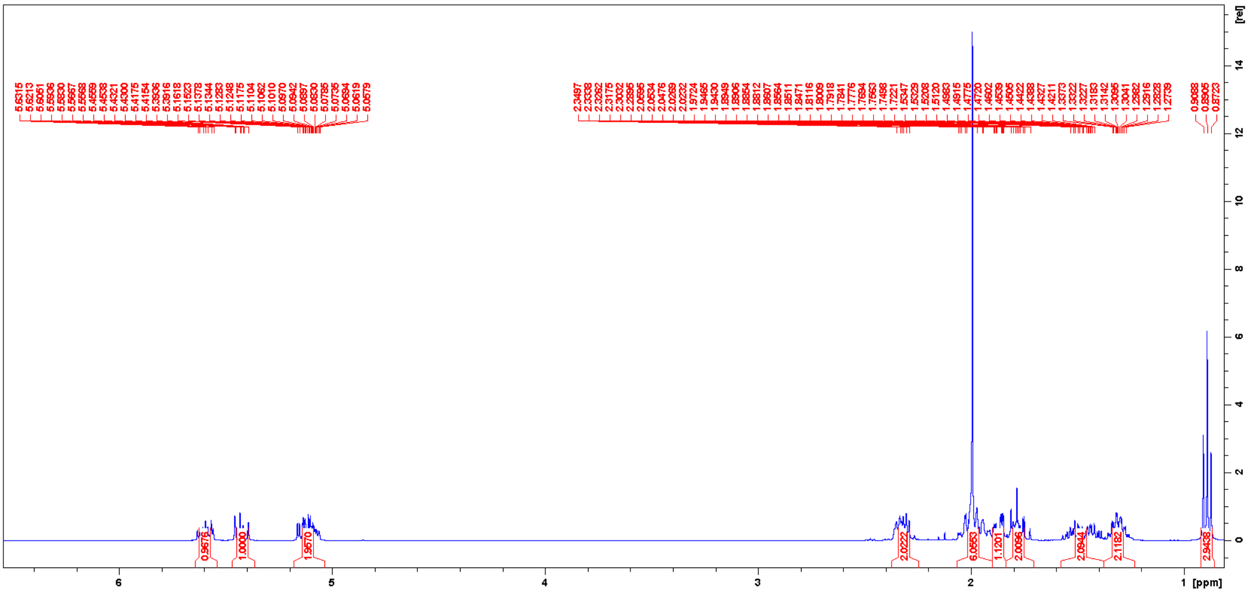

Figure S29 – ^{1}H NMR spectrum of compound **9**

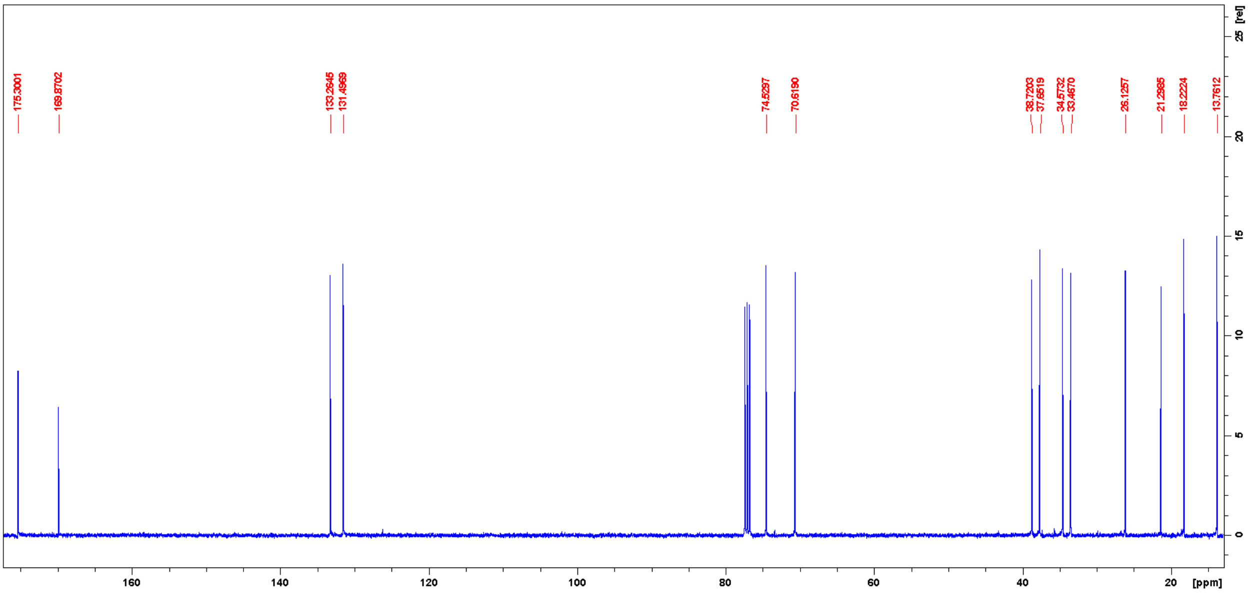

Figure S30 –^{13}C NMR spectrum of compound **9**

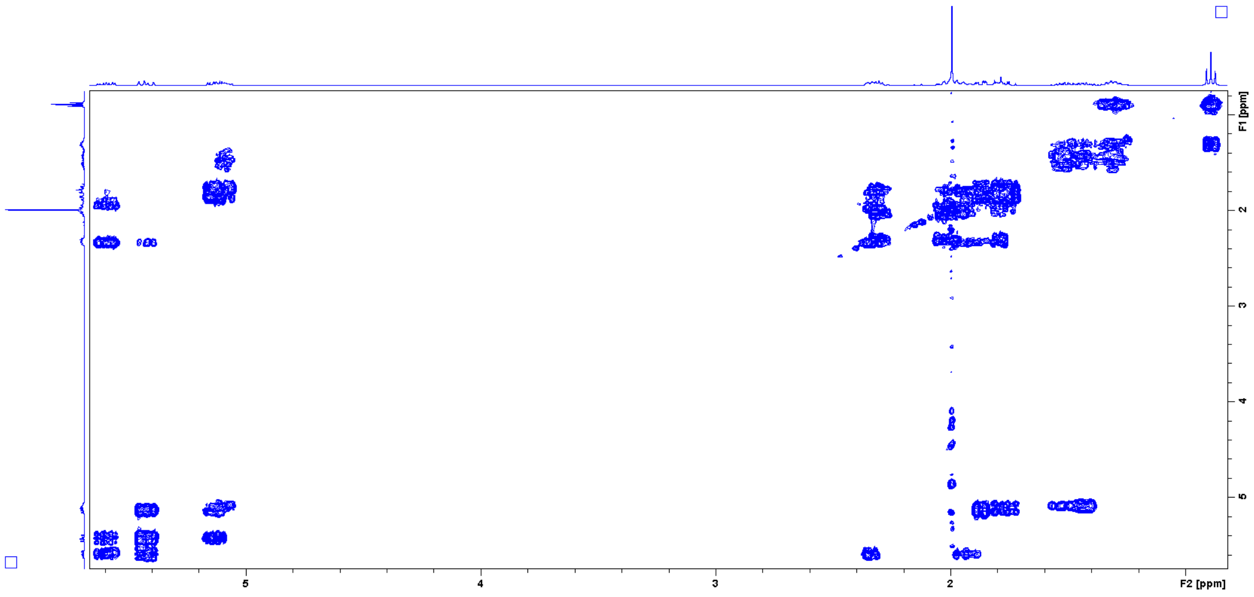

Figure S31 – COSY spectrum of compound **9**

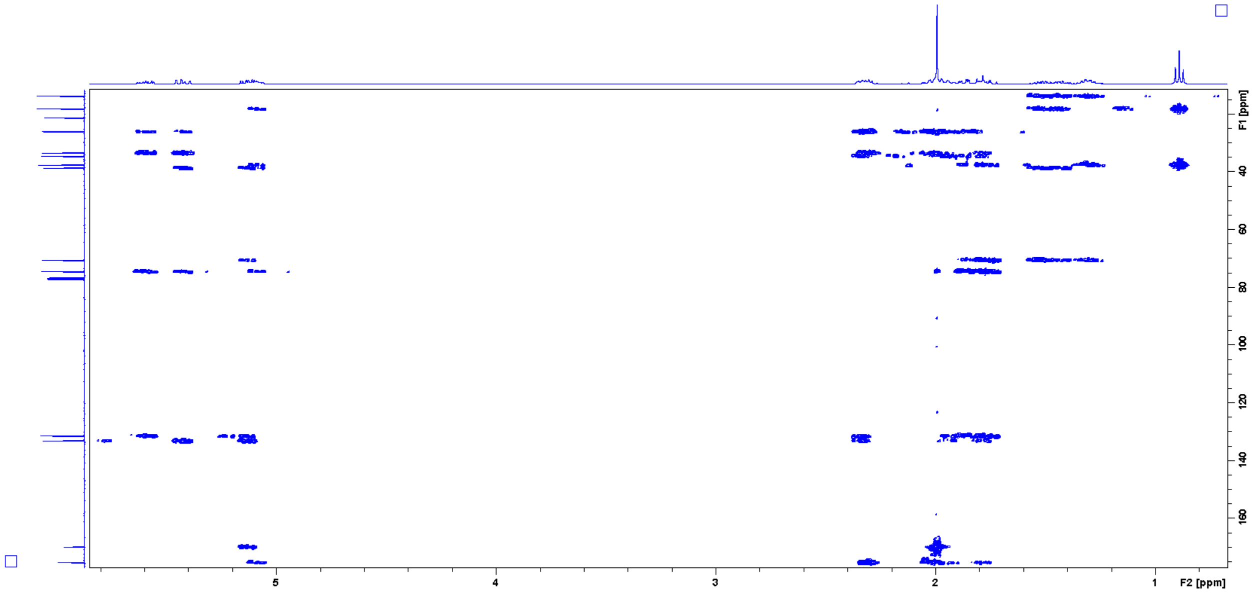

Figure S32 – HMBC spectrum of compound **9**

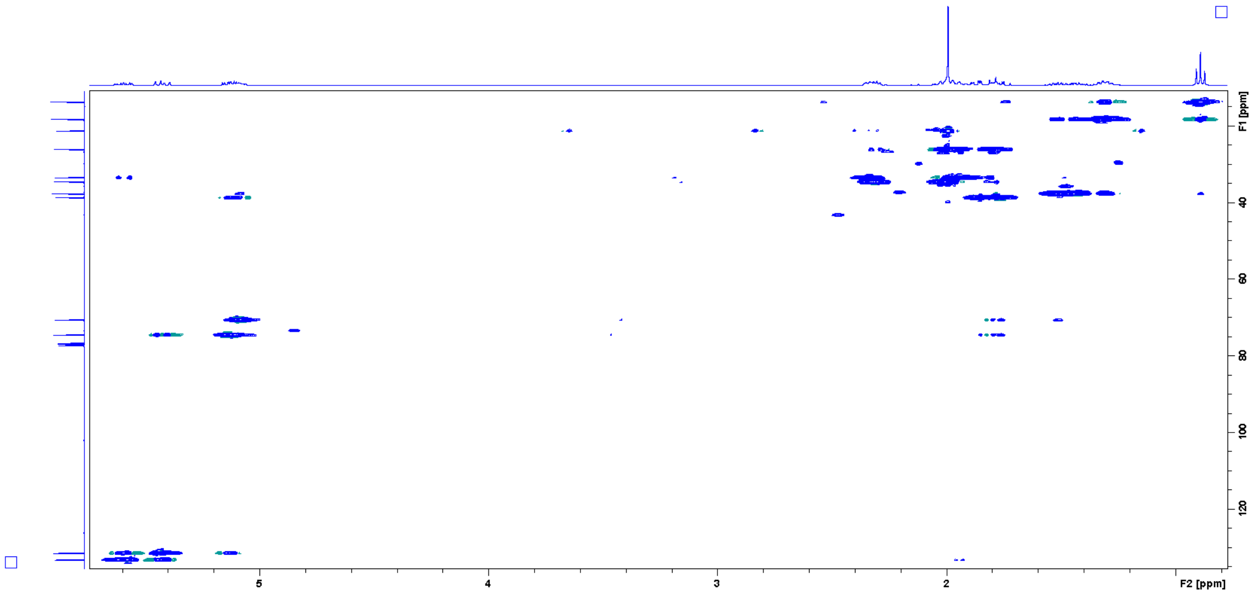

Figure S33 – HSQC spectrum of compound **9**

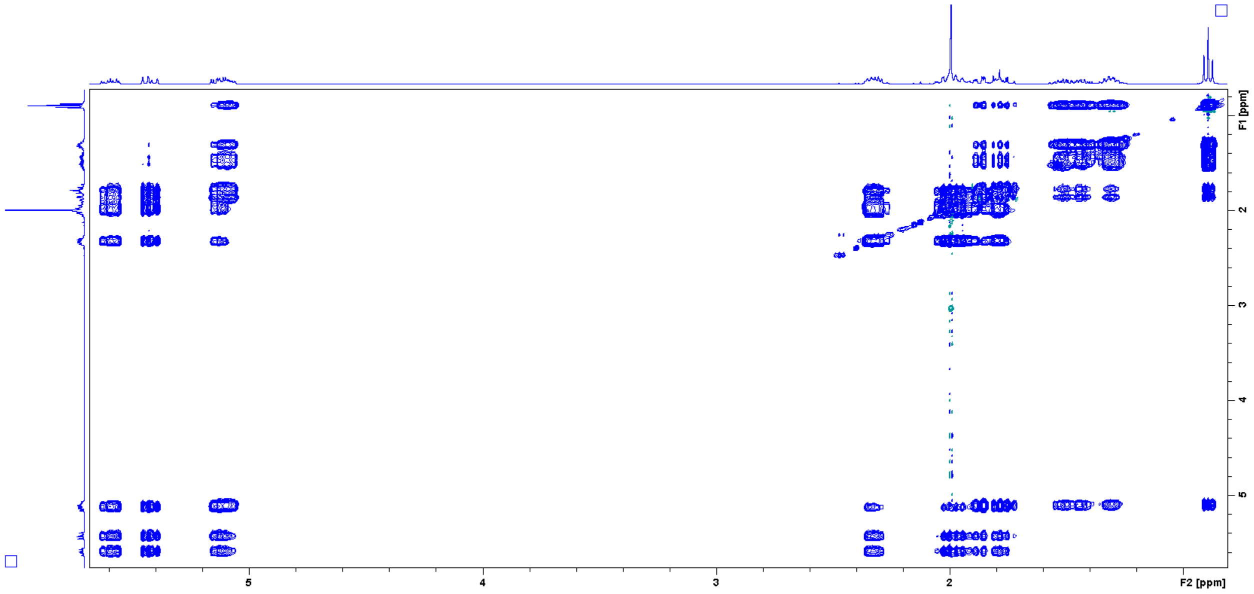

Figure S34 – TOCSY spectrum of compound **9**

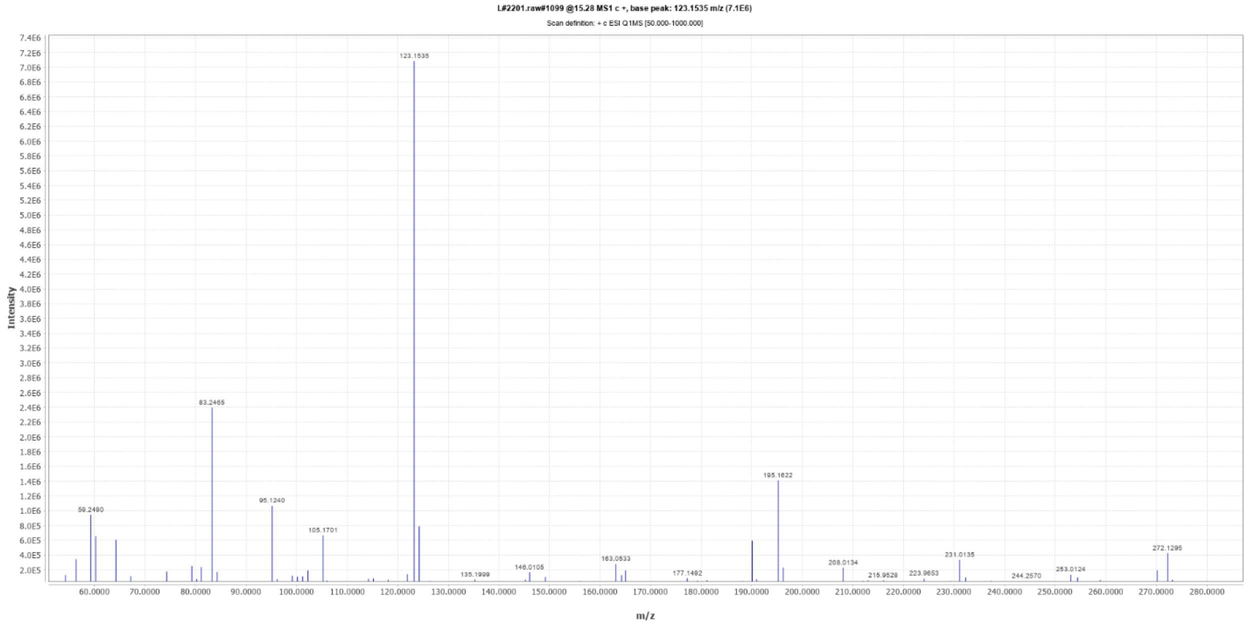

Figure S35 – ESIMS of compound **9** recorded in positive ion mode

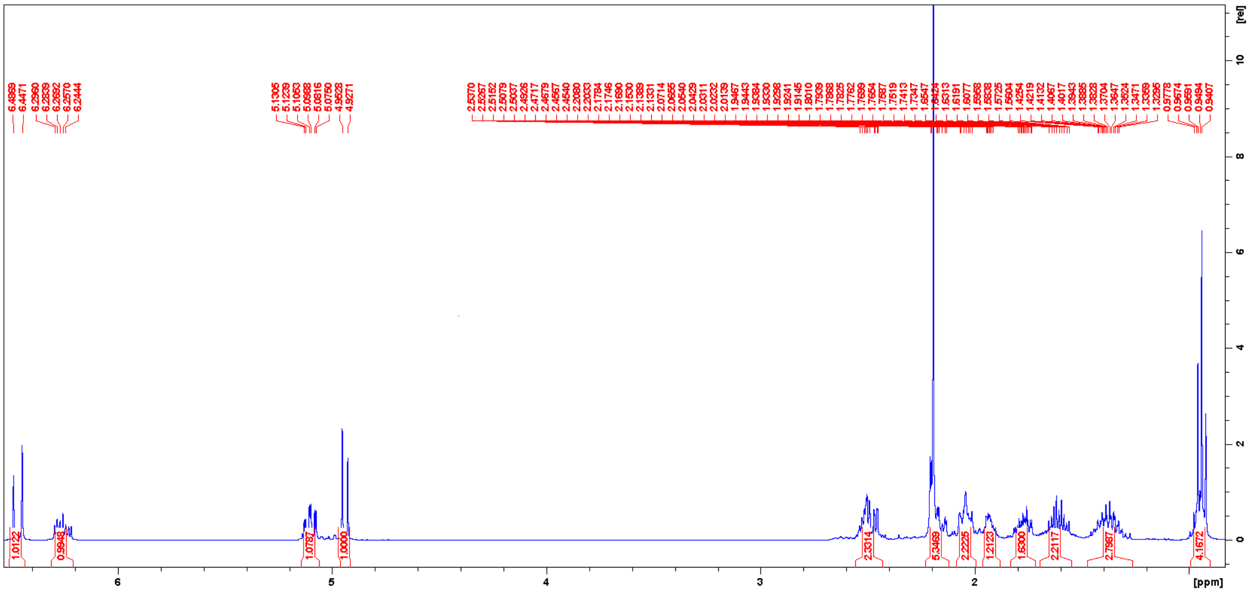

Figure S36 – ^{1}H NMR spectrum of compound **10**

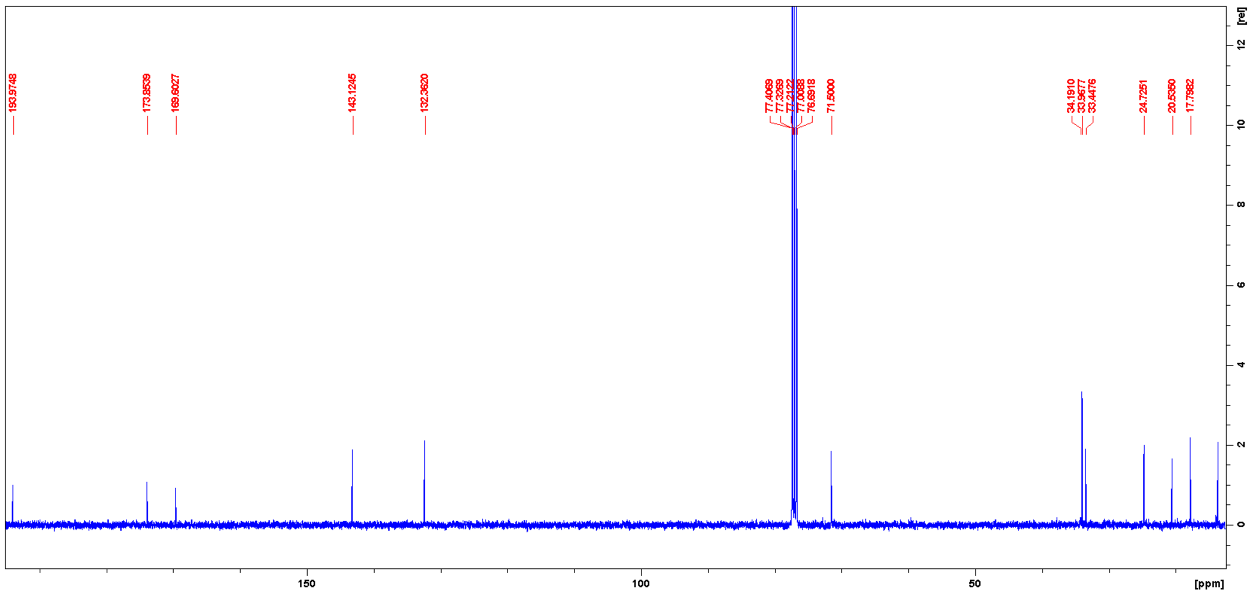

Figure S37 –^{13}C NMR spectrum of compound **10**

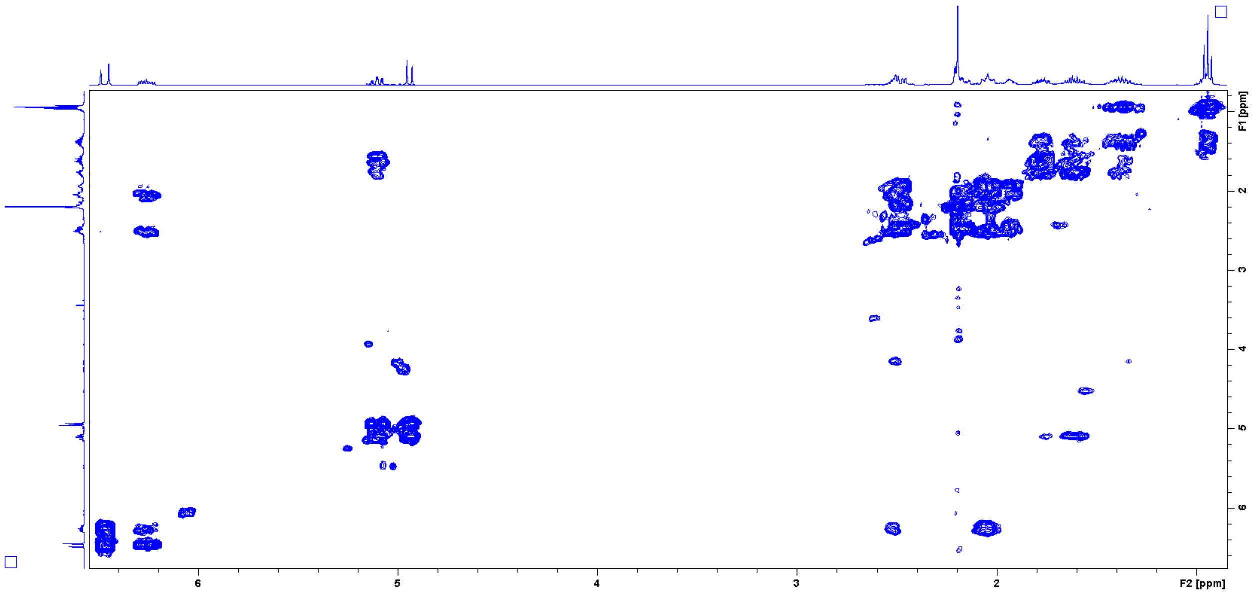

Figure S38 – COSY spectrum of compound **10**

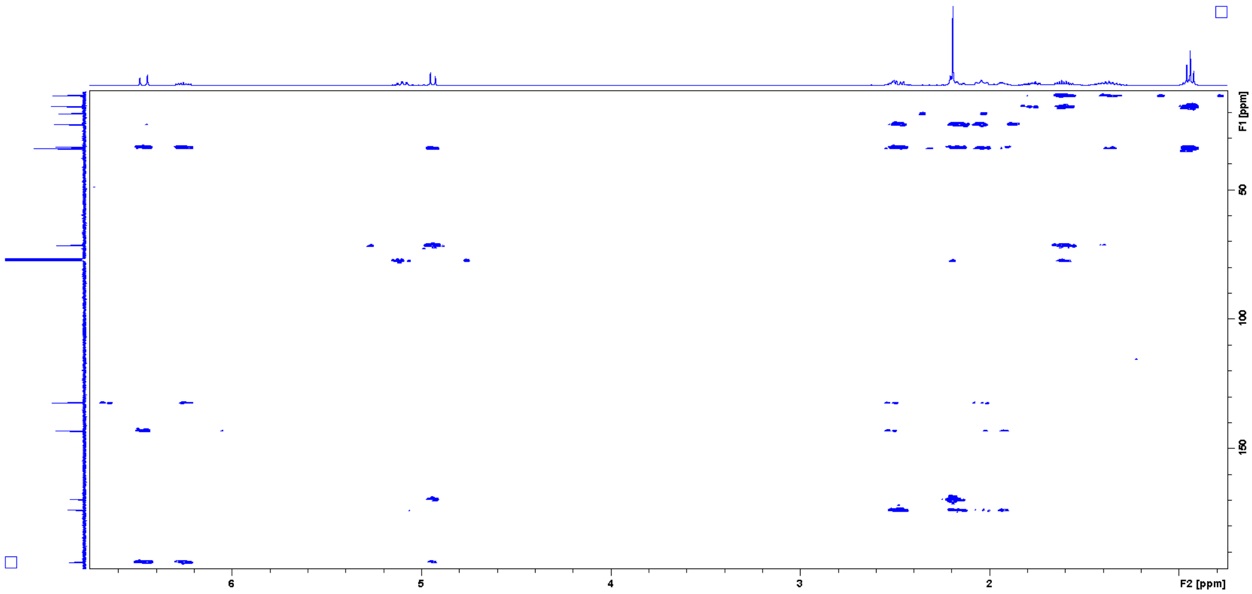

Figure S39 – HMBC spectrum of compound **10**

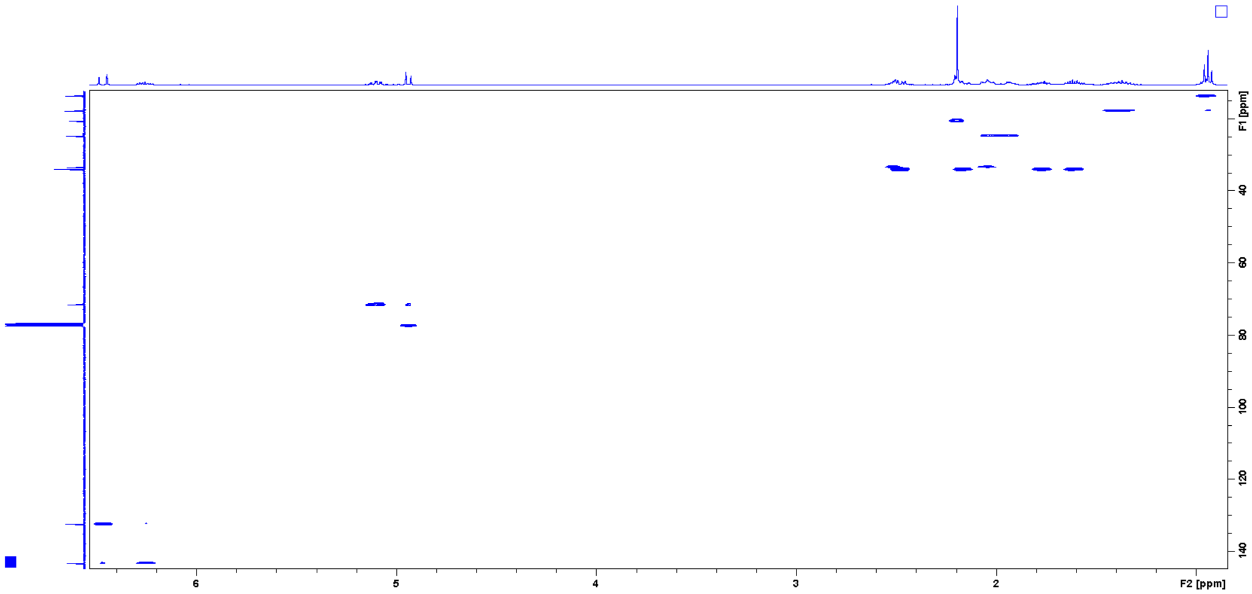

Figure S40 – HSQC spectrum of compound **10**

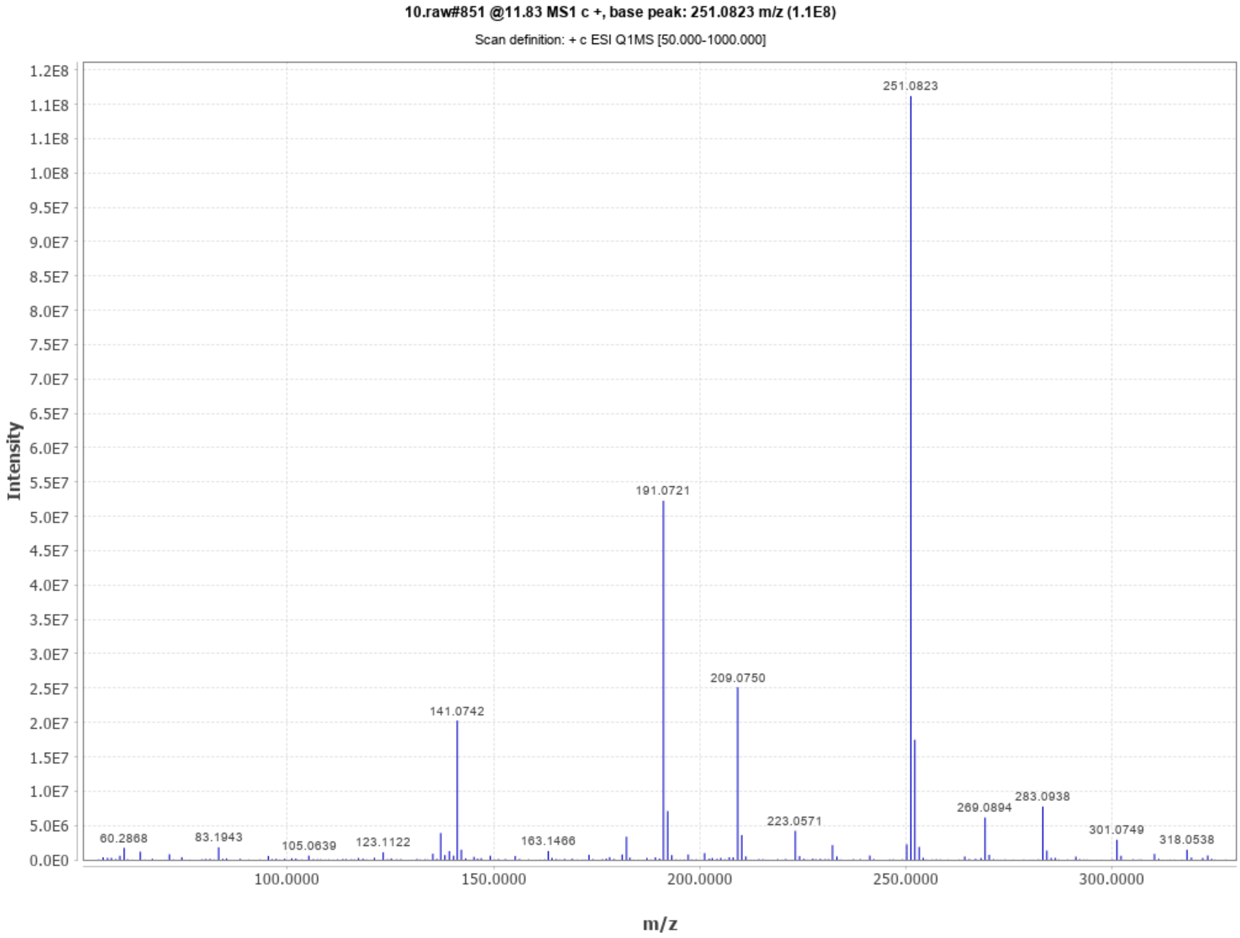

Figure S41 – ESIMS of compound **10** recorded in positive ion mode

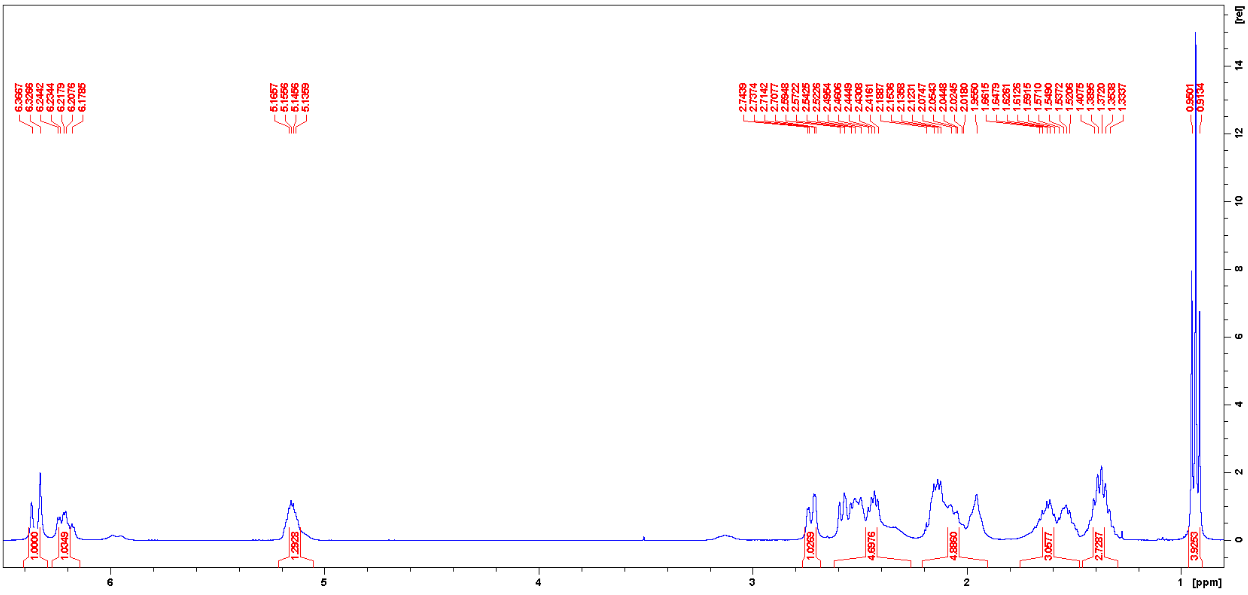

Figure S42 – ^{1}H NMR spectrum of compound **11**

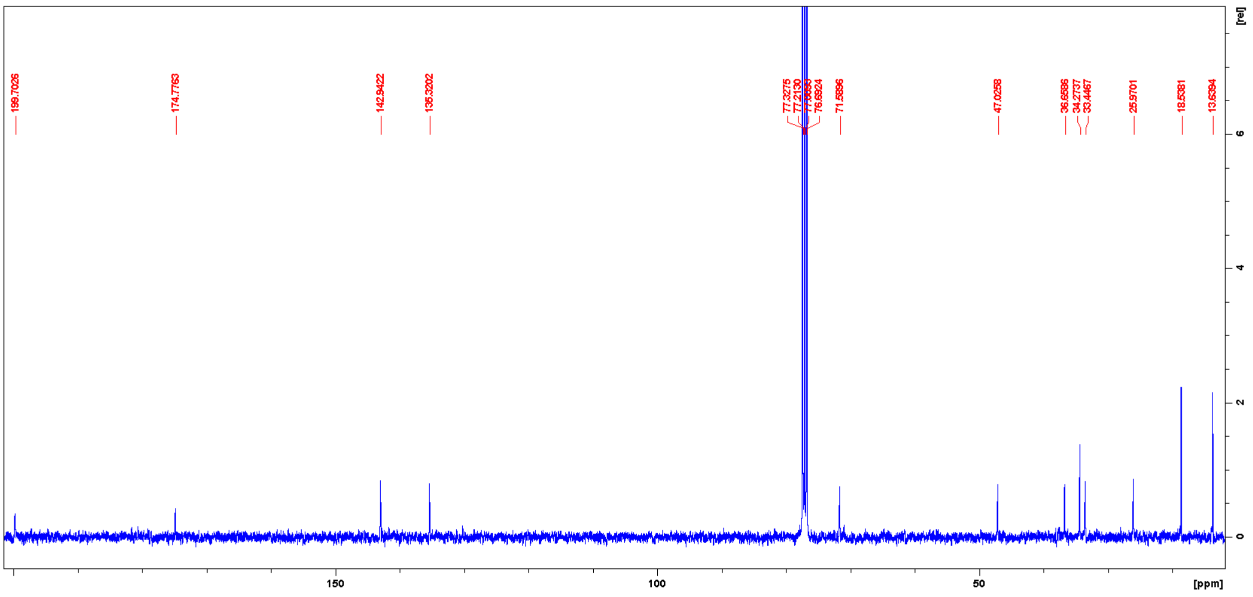

Figure S43 –^{13}C NMR spectrum of compound **11**

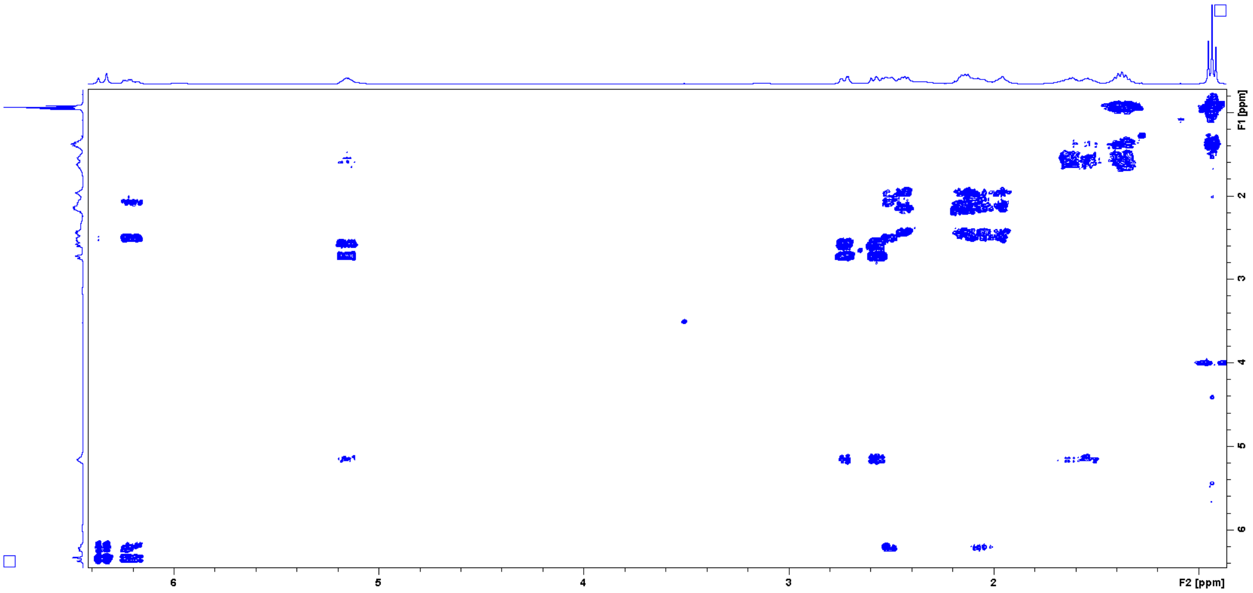

Figure S44 – COSY spectrum of compound **11**

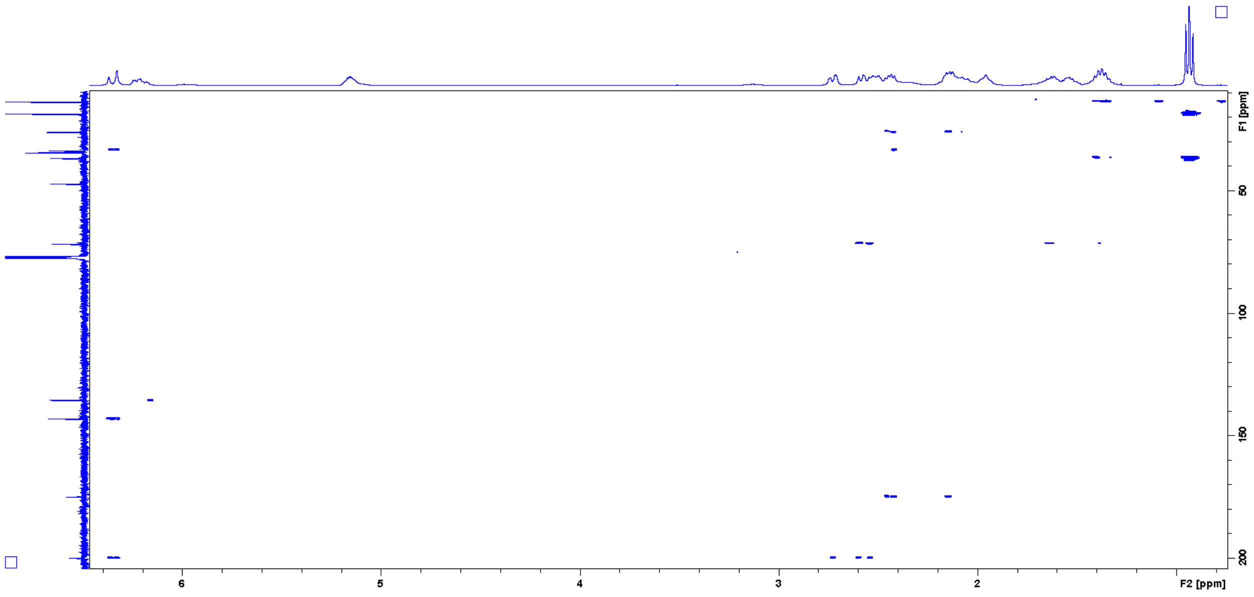

Figure S45 – HMBC spectrum of compound **11**

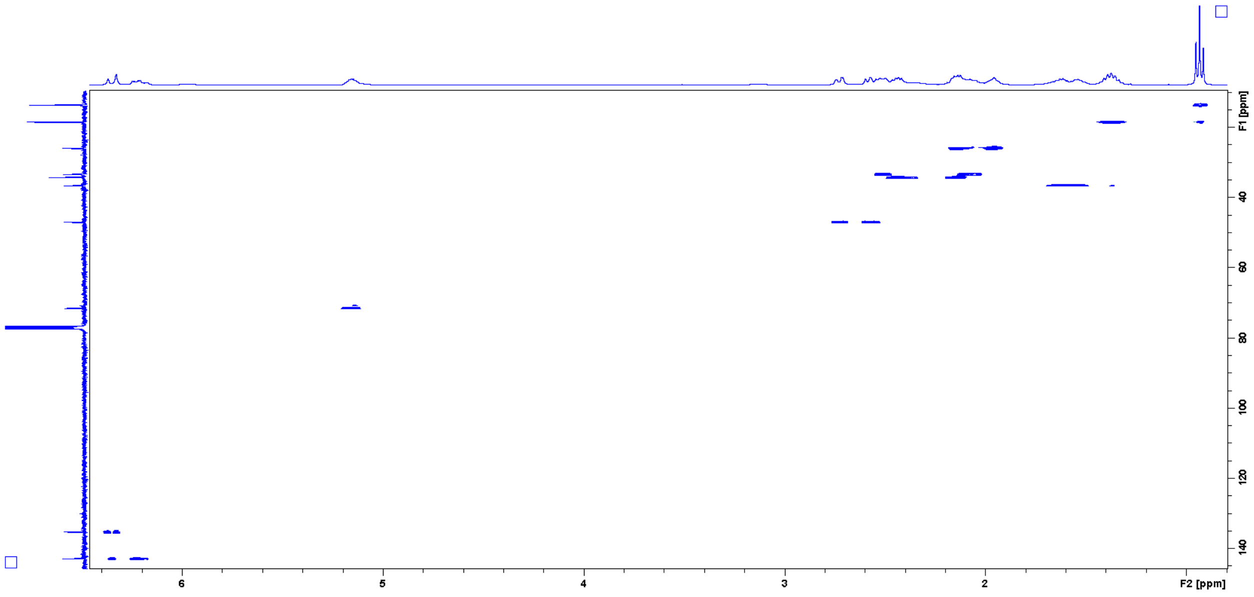

Figure S46 – HSQC spectrum of compound **11**

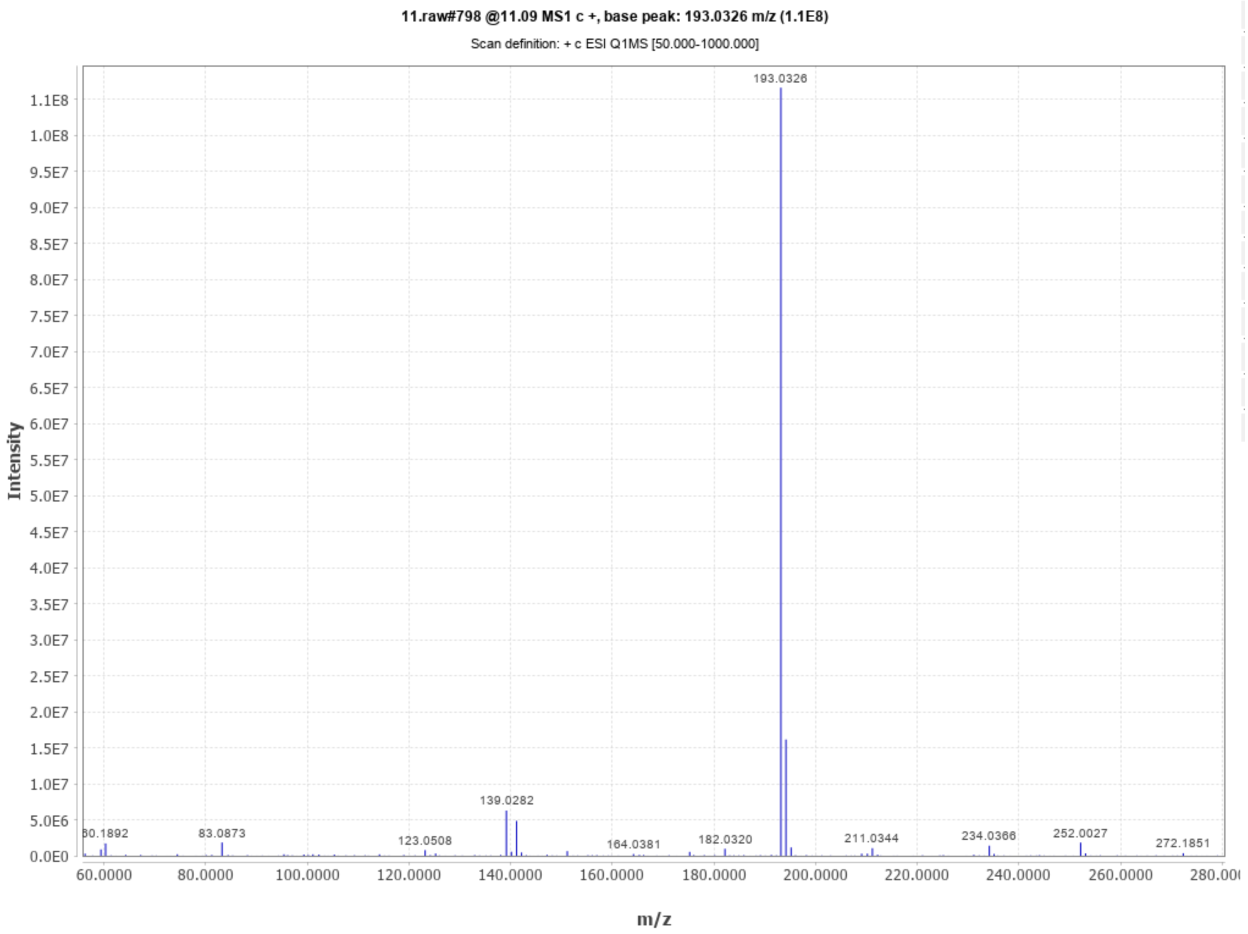

Figure S47 – ESIMS of compound **11** recorded in positive ion mode

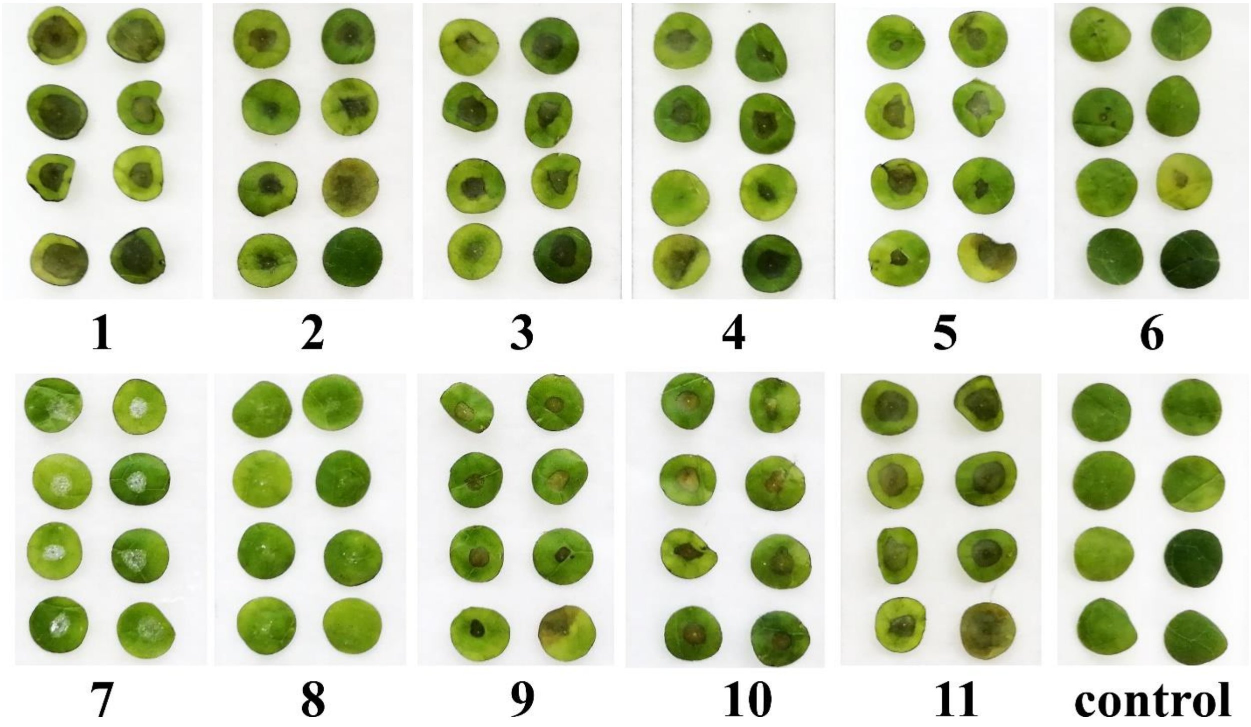

Figure S48 – Phytotoxic activity of compounds **1** – **11** on sowthistle punctured leaf discs at 120 hours after treatment
